# UDP‐glucose dehydrogenase expression is upregulated following EMT and differentially affects intracellular glycerophosphocholine and acetylaspartate levels in breast mesenchymal cell lines

**DOI:** 10.1002/1878-0261.13172

**Published:** 2022-02-03

**Authors:** Qiong Wang, Sigurdur Trausti Karvelsson, Freyr Johannsson, Arnar Ingi Vilhjalmsson, Lars Hagen, Davi de Miranda Fonseca, Animesh Sharma, Geir Slupphaug, Ottar Rolfsson

**Affiliations:** ^1^ Center for Systems Biology Biomedical Center Faculty of Medicine School of Health Sciences University of Iceland Reykjavik Iceland; ^2^ Department of Clinical and Molecular Medicine Norwegian University of Science and Technology NTNU Trondheim Norway; ^3^ Clinic of Laboratory Medicine St. Olavs Hospital Trondheim Norway; ^4^ PROMEC Core Facility for Proteomics and Modomics Norwegian University of Science and Technology, NTNU, and the Central Norway Regional Health Authority Norway Trondheim Norway

**Keywords:** acetylaspartate, breast cancer, EMT, glycerophosphocholine, PDGFRB, UGDH

## Abstract

Metabolic rewiring is one of the indispensable drivers of epithelial–mesenchymal transition (EMT) involved in breast cancer metastasis. In this study, we explored the metabolic changes during spontaneous EMT in three separately established breast EMT cell models using a proteomic approach supported by metabolomic analysis. We identified common proteomic changes, including the expression of CDH1, CDH2, VIM, LGALS1, SERPINE1, PKP3, ATP2A2, JUP, MTCH2, RPL26L1 and PLOD2. Consistently altered metabolic enzymes included the following: FDFT1, SORD, TSTA3 and UDP‐glucose dehydrogenase (UGDH). Of these, UGDH was most prominently altered and has previously been associated with breast cancer patient survival. siRNA‐mediated knock‐down of *UGDH* resulted in delayed cell proliferation and dampened invasive potential of mesenchymal cells and downregulated expression of the EMT transcription factor SNAI1. Metabolomic analysis revealed that siRNA‐mediated knock‐down of *UGDH* decreased intracellular glycerophosphocholine (GPC), whereas levels of acetylaspartate (NAA) increased. Finally, our data suggested that platelet‐derived growth factor receptor beta (PDGFRB) signalling was activated in mesenchymal cells. siRNA‐mediated knock‐down of *PDGFRB* downregulated *UGDH* expression, potentially via NFkB‐p65. Our results support an unexplored relationship between UGDH and GPC, both of which have previously been independently associated with breast cancer progression.

## Graphical abstract

Epithelial–mesenchymal transition (EMT) is hijacked by cancer cells during metastasis. Metabolic rewiring is an indispensable driver of EMT and cancer progression. In this study, we observed that UDP‐glucose dehydrogenase (UGDH) was upregulated following EMT. UGDH influenced SNAI1 expression and mesenchymal cell proliferation and invasion. Additionally, the intracellular levels of glycerophosphocholine and acetylaspartate were independently regulated by UGDH. In breast mesenchymal cell lines, UGDH expression was regulated by PDGFRB via NFkB‐p65.

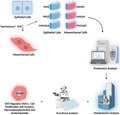

**Abbreviations**
AGC: automatic gain control
ATP2A2: sarcoplasmic/endoplasmic reticulum calcium ATPase 2
CD44: CD44 antigen
CDH1: E‐cadherin
CDH2: N‐cadherin
EGFR: epidermal growth factor receptor
EMT: epithelial–mesenchymal transition
FDFT1: squalene synthase
FDR: false discovery rate
FLNA: filamin‐A
FSCN1: fascin
GEMs: genome‐scale metabolic network reconstructions
GPC: glycerophosphocholine
HBP: hexosamine biosynthesis pathway
JUP: junction plakoglobin
LGALS1: galectin‐1
LMNB1: Lamin‐B1
MSN: moesin
MTCH2: mitochondrial carrier homolog 2
NAA: acetylaspartate
NDRG1: N‐myc downstream regulated 1
PDGFRB: platelet‐derived growth factor receptor beta
pERK: extracellular signal‐regulated kinase (phosphorylated)
PKP3: plakophilin‐3
PLA2G15: phospholipase A2 group XV
PLOD2: procollagen‐lysine,2‐oxoglutarate
PPARγ: peroxisome proliferator‐activated receptor γ
PSM: peptide‐spectrum matches
RELA (NFκB‐p65): nuclear factor NF‐kappa‐B p65 subunit
RPL26L1: 60S ribosomal protein L26‐like 1
S100A2: S100 calcium‐binding protein A2
SERPINE1: plasminogen activator inhibitor 1
SNAI1: Snail family transcriptional repressor 1
SORD: sorbitol dehydrogenase
TLN1: Talin‐1
TSTA3: GDP‐L‐fucose synthase
UDP‐Glc: UDP‐glucose
UDP‐GlcA: UDP‐glucuronate
UGDH: UDP‐glucose dehydrogenase
VIM: vimentin

## 1 Introduction

Epithelial–mesenchymal transition (EMT) is a core developmental process that allows a polarized epithelial cell to assume mesenchymal phenotypes through a series of morphological, molecular, regulatory and functional changes [1]. EMT is part of normal tissue development, organ/tissue fibrosis, wound healing and cancer malignancy. Partial activation of EMT drives tumour metastasis and dissemination to distant organs [2, 3].

The biological plasticity and molecular heterogeneity of the EMT programme indicate that EMT is context‐specific, which has resulted in discrepancies in its description in the literature [4]. One factor causing EMT heterogeneity in cell line models is the EMT induction method. Growth factors, transcription factors and microRNAs can be manipulated in cells to trigger EMT, such as TGF‐β, EGF, Snail1/2, ZEB1 and Twist [3, 5, 6, 7, 8]. Genetic manipulation may lead to effects that diminish the flexibility and plasticity of the EMT programme. In the present study, we used three breast EMT cell models related to basal mammary cells to investigate common effects of the spontaneous EMT programme, that is, D492 EMT cell lines [9, 10], HMLE EMT cell lines [7, 11, 12] and PMC42 EMT cell lines [13, 14, 15]. Each EMT cell model includes a breast epithelial/mesenchymal cell line pair generated with spontaneous induction methods without overexpressing specific EMT inducers. These EMT models may reside at different positions in the EMT spectrum [1] but represent typical EMT progress in human breast gland development and have contributed significantly to the understanding of the molecular regulatory machinery in EMT [16, 17, 18].

Metabolic reprogramming is an indispensable driver of EMT in cancer [19]. A better understanding of the metabolism of EMT may facilitate the development of new therapeutics for breast cancer treatment. Glucose metabolism, lipid metabolism, an acidic microenvironment, nucleotide metabolism and amino acid metabolism have been related to EMT in cancer malignancy [20, 21]. In our previous studies on EMT‐related metabolic dysregulation limited to the D492 EMT model, we observed different preferences for reductive/oxidative carboxylation, glycolysis and amino acid anaplerosis along with an altered lipid profile and shifted glutathione homeostasis [22, 23, 24]. To move towards a system's understanding of how metabolism is influenced following EMT, we compared the metabolic phenotypes of EMT within genome‐scale metabolic network reconstructions (GEMs) that allow the integrated analysis of gene expression, proteomic and metabolomic data. These analyses revealed increased dependency on argininosuccinate lyase (ASL) and enhanced activities of the pentose phosphate pathway, hexosamine biosynthesis and one‐carbon metabolism post‐EMT in the D492 EMT model [23, 25]. More recently, we confirmed that metabolic flux through the hexosamine biosynthesis pathway (HBP) increases significantly in mesenchymal cells and that glutamine‐fructose‐6‐phosphate transaminase 2 (GFPT2) in the HBP is associated with breast cancer malignancy [26].

**Fig. 1:**
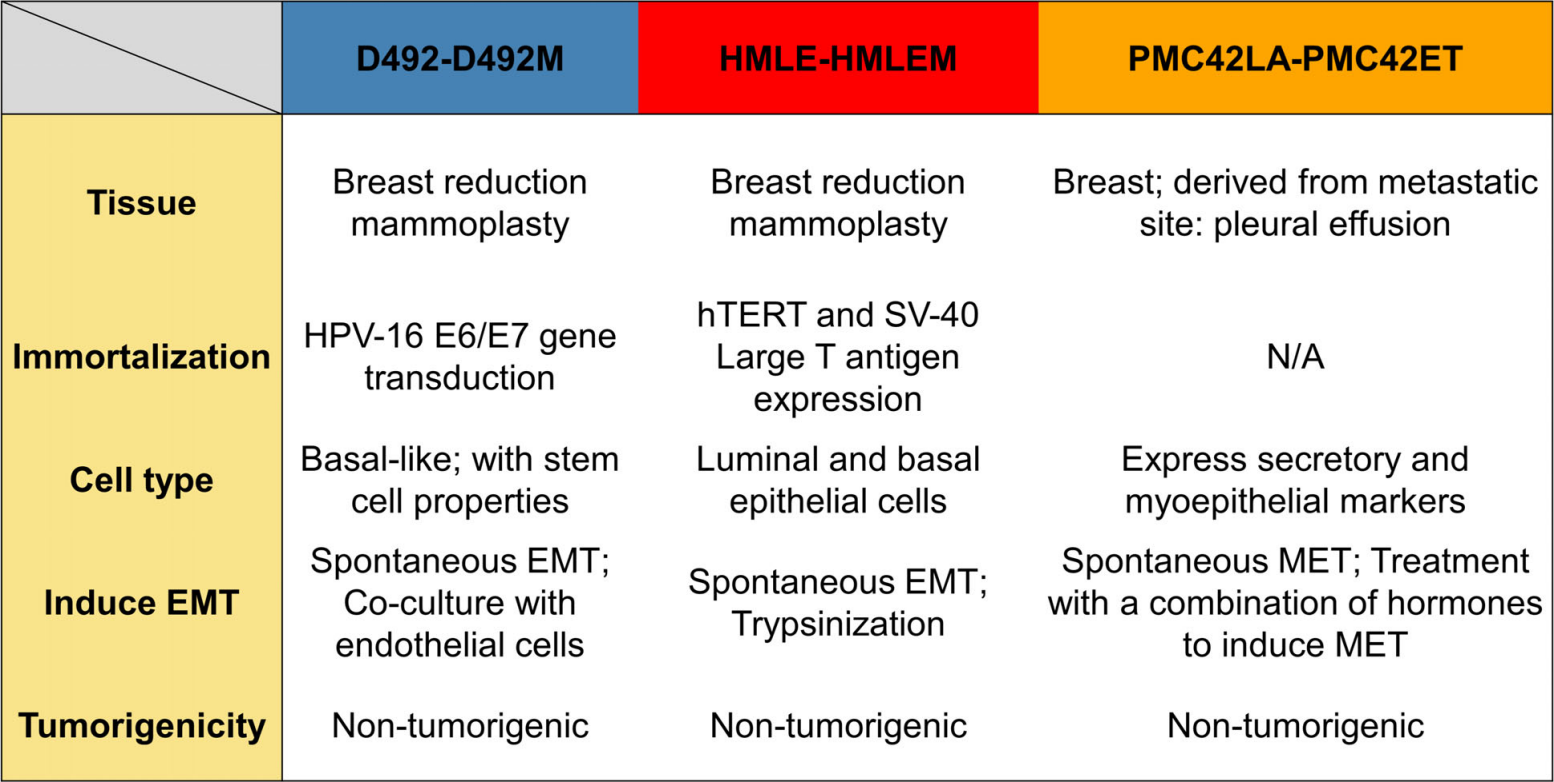
Summary of the three breast EMT cell models. Comparison of the three breast EMT cell models with respect to tissue origin, immortalization methods, cell markers, EMT induction methods and tumorigenicity.

In this study, we further explored the metabolic changes in EMT using shotgun proteomics and expanded our analysis to include three breast cell models descriptive of spontaneous EMT (Fig. 1 and Fig. S1). Several metabolic enzymes were commonly changed after EMT, with UDP‐glucose dehydrogenase (UGDH) being most altered. UGDH catalyses conversion of UDP‐glucose (UDP‐Glc) to UDP‐glucuronate (UDP‐GlcA), both of which are essential metabolites with diverse cellular functions [27, 28]. UGDH is involved in a variety of regulatory events. SP1, TGF‐β, Slit2, p38^MAPK^ and PI3K/Akt regulate UGDH expression, which in turn influences the downstream targets ERK/MAPK, PPARγ and SNAI1 [27, 28, 29, 30, 31, 32, 33, 34]. Several studies have recently reported that UGDH is involved in tumour growth, metastasis and patient survival [27, 32, 34, 35, 36, 37]. To understand the roles of UGDH in EMT in the breast gland, we knocked down *UGDH* in breast mesenchymal cells with siRNAs and studied effects on cell function and metabolism. Importantly, the three EMT models studied are noncarcinogenic. To account for UGDH in cancer progression and oncogenesis, we compared results from the EMT models to UGDH functions in the tumorigenic breast mesenchymal cell lines D492HER2 and MDA‐MB‐231. These investigations suggest that the tumour promoting effects of UGDH may in part be attributed to changes in choline metabolism.

## 2 Materials and methods

### 2.1 Cell culture

D492 was isolated from primary cultures of reduction mammoplasties with immortalization [9]. D492M was generated via 3D coculture of the D492 cells with endothelial cells to induce EMT [10]. D492HER2 was established by the overexpression of HER2 receptors on D492 [38]. The D492 cell lines (D492, D492M and D492HER2) were cultured in serum‐free H14 medium (DMEM/F‐12 without glutamine; Thermo Fisher Scientific (TFS), Waltham, MA, USA; 21331020) supplemented with 250 ng·mL^−1^ insulin (Merck, Kenilworth, NJ, USA; I6634), 10 µg·mL^−1^ transferrin (Merck; T2252), 10 ng·mL^−1^ EGF (PeproTech, Cranbury, NJ, USA; AF‐100‐15), 2.6 ng·mL^−1^ Na‐selenite (BD Biosciences, San Jose, CA, USA; 354201), 10^−10^ m estradiol (Sigma, St.Louis, MO, USA; E2758), 1.4 × 10^−6^ m hydrocortisone (Sigma; H0888), 0.15 IU prolactin (PeproTech; 100‐07), 100 IU penicillin and 0.1 mg·mL^−1^ streptomycin (Gibco™, TFS; 15140122) and 2 mm glutamine (TFS; 25030024). The passage numbers for both D492 and D492M were from 31, while D492HER2 was cultured from passage 65. HMLE was isolated from reduction mammoplasties [12], while HMLEM was generated from HMLE via differential trypsinization [7]. The HMLE cell lines (HMLE with passage number from 16 and HMLEM with passage number from 28) were cultured in serum‐free DMEM/F‐12 medium supplemented with 10 µg·mL^−1^ insulin, 10 ng·mL^−1^ EGF, 1.4 × 10^−6^ m hydrocortisone, 100 IU penicillin and 0.1 mg·mL^−1^ streptomycin and 2 mm glutamine. PMC42ET was originally established from pleural effusion from the metastatic site in a breast cancer patient [15], and PMC42LA was then generated via mesenchymal–epithelial transition by hormone treatments [13]. The PMC42 cell lines (PMC42LA and PMC42ET with passage numbers from 9) were cultured in RPMI 1640 Medium (TFS; 52400025) supplemented with 10% FBS (Gibco™ 10270106) and 100 IU penicillin and 0.1 mg·mL^−1^ streptomycin. The antibiotics were excluded in the medium for the transient knock‐down experiments. In the SILAC phosphoproteomic experiment, DMEM/F‐12 was replaced by ‘DMEM:F‐12 for SILAC’ (TFS; 88370) with light‐ (l‐arginine, l‐lysine), medium‐ [l‐arginine‐^13^C_6_ hydrochloride (Arg + 6 Da), l‐lysine‐4,4,5,5‐d4 hydrochloride (Lys + 4 Da)] or heavy‐ (l‐arginine‐^13^C_6_,^15^N_4_ hydrochloride (Arg + 10 Da), l‐lysine‐^13^C_6_,^15^N_2_ hydrochloride (Lys + 8 Da)] labelled arginine or lysine (Cambridge Isotope Laboratories, Tewksbury, MA, USA). In the invasion assay, H14 was supplemented with 10% FBS in the lower chamber of the Transwell. The MDA‐MB‐231 cells (passage number 26) were cultured in RPMI 1640 supplemented with 10% FBS and 100 IU penicillin and 0.1 mg·mL^−1^ streptomycin. All cell lines were cultured at 37 °C with 5% CO_2_ for routine maintenance, and cells were routinely checked for mycoplasma contamination. All cell lines used in this study were kindly provided by the Stem Cell Research Unit, Biomedical Center, University of Iceland.

### 2.2 LFQ proteomics and SILAC phosphoproteomic analysis

The proteomic experimental set‐up was illustrated in Fig. S1A.

#### 2.2.1 LFQ protein and peptide sample preparation

Cells were lysed with 4% sodium dodecyl sulfate (SDS; MP Biomedicals™, Irvine, CA, USA) in 100 mm Tris (Sigma) and kept on ice for 10 min and then transferred to 1.5‐mL Eppendorf tubes. After five freeze/thaw (−80 °C/room temperature) cycles, the samples were centrifuged at 20 817 ***g*** for 20 min at 4 °C. The supernatants were collected and aliquoted in new tubes and stored at −80 °C. Total protein was quantified with the Pierce™ BCA protein assay (TFS). A volume containing 12–15 µg total protein was precipitated by chloroform/methanol precipitation and reconstituted in 50 mm ammonium bicarbonate. The protein sample was reduced with 1 m dithiothreitol (DTT) for 20 min at 70 °C and then alkylated by 200 mm iodoacetamide (IAM) at room temperature in the dark for 30 min, followed by quenching the extra IAM with 1 m DTT for 20 min at room temperature in the dark. Samples were digested overnight with 1.5 μg trypsin at 37 °C. Tryptic peptides were desalted using C‐18 StageTips as described [39], after which peptides were dried in a SpeedVac centrifuge and resuspended in 0.1% formic acid.

#### 2.2.2 LFQ LC‐MS/MS analysis

Peptides were analysed on an LC‐MS/MS platform consisting of an Easy‐nLC 1200 UHPLC system (TFS) interfaced with a QExactive HF Orbitrap Mass Spectrometer (TFS) via a nanospray ESI ion source (TFS). Peptides were injected into a C‐18 trap column (Acclaim PepMap100, 75 μm i. d. × 2 cm, C‐18, 3 μm, 100 Å; TFS) and further separated on a C‐18 analytical column (Acclaim PepMap100, 75 μm i. d. × 50 cm, C‐18, 2 μm, 100 Å; TFS) using a multistep gradient with buffer A (0.1% formic acid) and buffer B (80% CH_3_CN, 0.1% formic acid): from 2% to 10% buffer B in 10 min, 10% to 50% buffer B in 130 min, 50% to 100% buffer B in 20 min and 20 min with 100% buffer B. The HPLC was re‐equilibrated with 2% buffer B before the next injection. The flow rate was 250 nL·min^−1^. Peptides eluted were analysed on QExactive HF mass spectrometer (TFS) operating in positive ion‐ and data‐dependent acquisition mode using the following parameters: electrospray voltage 1.9 kV, HCD fragmentation with normalized collision energy 29, automatic gain control (AGC) target value of 3 × 10^6^ for Orbitrap MS and 1 × 10^5^ for MS/MS scans. Each MS scan (*m/z* 350–1650) was acquired at a resolution of 120 000 FWHM, followed by 15 MS/MS scans triggered for AGC targets above 2 × 10^3^, at a maximum ion injection time of 100 ms for MS and 100 ms for MS/MS scans. The proteomic method has been described previously [40].

#### 2.2.3 LFQ protein and peptide identification and quantification

Proteins were identified and quantified by processing MS data using Thermo Scientific™ proteome discoverer™ (PD, version 2.3; TFS). preview version 2.3.5 (Protein Metrics Inc.) [41] was used to inspect the raw files to determine optimal search criteria, and the following search parameters were used: (a) enzyme specified as trypsin with maximum of two missed cleavages allowed; (b) acetylation of protein N‐terminal including loss‐of‐methionine; (c) oxidation of methionine; (d) deamidation of asparagine/glutamine as dynamic post‐translational modification; (e) carbamidomethylation of cysteine as static; Precursor mass‐tolerance of 10 PPM while fragment mass‐tolerance of 0.02 Dalton. PD's node, Spectrum file RC, was set up to query the raw files against the human proteome downloaded from UniProt (*Homo sapiens*, UP000005640, October 2018) with the static modification to recalibrate and detect features with the Minora node. Further, the internal contaminant database was also queried along with the human proteome using Sequest [42] search engine available in PD. For downstream analysis of these peptide‐spectrum matches (PSM), both protein and peptide identifications/PSM false discovery rate (FDR) were set to 1%; thus, only the unique peptides with high confidence were used for final protein group identification. Peak abundances were extracted by integrating the area under the peak curve. Each protein group abundance was normalized by the total abundance of all identified peptides at FDR < 1%. Summed up median values for all unique peptide ion abundances mapped to respective protein using label‐free quantification scaled on all average with Precursor Ion Quantifier node [43] for PD were used.

The mass spectrometry proteomic data have been deposited to the ProteomeXchange Consortium via the PRIDE [44] partner repository with the data set identifier PXD024164.

The protocol for SILAC phosphoproteomic analysis was thoroughly described in the other study [26]. Briefly, protein sample equivalent to 4 mg was dissolved and fractionated, followed by phosphorylated peptide enrichment with MagReSyn‐TiIMAC beads (Resyn Biosciences, Edenvale, Gauteng, South Africa) and Magnetic Rack (DynaMag‐2; Life Technologies, Carlsbad, CA, USA). Analyses of peptides for total proteome and phosphorylated proteome were carried out on a Velos‐Pro Orbitrap (TFS) mass spectrometer coupled with a Dionex UltiMate 3000 RS (TFS). The raw data files obtained from the mass spectrometric outputs for each experiment were merged into a single quantitated data set using maxquant (version 1.5.2.8) [45] and the andromeda search engine software [46]. The mass spectrometry phosphoproteomic data have been deposited to the ProteomeXchange Consortium via the PRIDE [44] partner repository with the data set identifier PXD025858.

### 2.3 Metabolomic analysis

The metabolomic experimental set‐up was illustrated in Fig. S1A. Cells were washed with saline solution (0.9%), and the metabolites were extracted with MeOH : dH_2_O (80 : 20) containing an internal standard mix (Table S1). After adding MeOH : dH_2_O (80 : 20), samples were centrifuged, and the supernatant was taken and vacuum dried. The extracts were analysed on UPLC mass spectrometry (SYNAPT G2; Waters) according to published protocols [47]. Metabolites were identified and quantified in masslynx software (version 4.2) from waters. For untargeted data analysis, xcms [48] was used for automatic peak‐picking (centWave) [49] and retention time alignment (OBI‐Warp) [50]. All features that eluted in the first 66 s were omitted from further analysis. Feature intensities were normalized using quality control sample‐based robust LOESS (locally estimated scatterplot smoothing) signal correction (QC‐RLSC) [51] which was implemented using the r‐package *NormalizeMets* [52]. For quality assurance, all features with over 25% relative standard deviation in the QC samples were omitted from further analysis. Generalized logarithmic transformation (glog) [53] and autoscaling were used to obtain mean‐centred, normally distributed feature intensity values with equal variance. The expression of metabolites was normalized to cell numbers estimated by crystal violet assays. For the normalization of the metabolic measurements in the metabolomic experiment, cells were counted using a crystal violet assay. In short, cells were fixed with 100% cold MeOH and stained with 0.25% crystal violet (Merck; C.I. 42555). After washing, stained cells were dissolved into 100 µL of 10% acetic acid and measured at 570 nm in the microplate reader (SpectraMax^®^ M3; Molecular Devices LLC, San Jose, CA, USA).

### 2.4 siRNA transient knock‐down and quantitative reverse transcription PCR

Cells were seeded either at 60 000 cells/well in 48‐well plates or at 480 000 cells/well in 6‐well plates. Before cell seeding, plates were coated with respective control siRNA (Silencer™ Select Negative Control, 4390843) and target siRNA (Silencer™ Select siUGDH: s409 and s410; siPDGFRB: s10240; siRELA: s11914 and s11915) as well as Lipofectamine™ RNAiMAX Transfection Reagent (TFS). Cells were transfected at 37 °C and 5% CO_2_ for 48 h with the final siRNA concentration of 10 nm.

In the RT‐qPCR experiments, cells were cultured in 48‐well plates for 72 h, followed by total RNA extraction with TRI Reagent™ Solution (Invitrogen™, TFS). RNA concentration was determined in NanoDrop One (TFS). 500–1000 ng of RNA was used for cDNA synthesis on the thermal cycler (Peltier Thermal Cycler, MJ research, PTC‐225, Alameda, CA, USA) using High‐Capacity cDNA Reverse Transcription Kit (TFS). Gene expression was measured with SYBR Green (Luna^®^ Universal qPCR Master Mix; New England BioLabs, Ipswich, MA, USA) on Bio‐Rad CFX384 Touch™ Real‐Time PCR Detection System (Bio‐Rad, Hercules, CA, USA). Primers were either selected from PrimerBank, designed on the Primer3Plus website, or based on the literature. Primer sequences for genes studied in this study were listed in Table S2.

### 2.5 Cell proliferation assay

Cells in quadruplicate were seeded at 10 000 cells/well in 96‐well plates. *UGDH* knock‐down followed the methods described above. For D492M, 24 h after seeding (48 h for D492HER2), cells were placed under the microscope (LEICA CTR 6500, bright field, 10×) at 37 °C with 5% CO_2_ for real‐time monitoring and multiple data acquisition. The microscope was controlled by software micro‐manager (version 1.4.22, Vale’s laboratory, San Francisco, CA, USA). Three spots were chosen in each well, and photographs were taken every 6 h. Cell growth was monitored for 66 h for D492M while 42 h for D492HER2. Photographs were batch‐processed with Macro in software imagej 1.52p (NIH, Bethesda, MD, USA), and cell numbers were normalized to the starting point.

### 2.6 Transwell invasion assay

The D492M and D492HER2 cells were cultured with siRNA transfection (Scramble and siUGDH) for 48 h in 6‐well plates. *UGDH* knock‐down followed the methods described above. Cells were then reseeded into filter units (Falcon^®^ Permeable Support for 24‐well Plate with 8.0‐µm Transparent PET Membrane, 353097, Corning, NY, USA) coated with Matrigel (Corning^®^ Matrigel^®^ Matrix, 356234) at a density of 30 000 cells/well. First, the filter inserts were coated with 100 µL 1 : 10 diluted Matrigel for 20–30 min at 37 °C. Next, 300 µL of cell suspension was added on top of the filter units. Then, 500 µL of H14 medium with 10% FBS was added to the wells in the 24‐well plates below the filters. Finally, cells were incubated at 37 °C and 5% CO_2_ for 48 h. Noninvasive cells on top of the filters were removed with cotton swabs, followed by fixation with paraformaldehyde (PFA, 3.7%, Sigma; 252549) and DAPI staining (1 : 5000; Sigma, D9542). Ten images per filter unit were taken by the EVOS^®^ FL Auto Imaging System (10×; TFS), followed by the batch analysis of the images in Macro imagej 1.52p. For normalization of the different cell numbers in the filter units, cells were seeded into a 24‐well plate along with the filter units and cultured and treated in the same way as cells in the filter units.

### 2.7 Statistical analysis and bioinformatics

All experiments performed in this study were in at least triplicates. The metabolomic analysis of the *UGDH* knock‐down treatment was in six replicates. The proteomic data were processed in perseus (version 1.6.14.0, data imputation based on normal distribution, width = 0.3, downshift = 1.8, permutation‐based FDR < 0.05) [54] and r (version 4.0.0, the University of Auckland, New Zealand). Plots in this study were generated in r software. The statistical significance for all two‐sample comparisons was based on the two‐sided Student's *t*‐test (Welsch, *P* < 0.05). Gene Ontology (GO) functional annotation was conducted in DAVID (DAVID Bioinformatics Resources 6.8) with default settings [55, 56]. Reactome pathway analysis was performed with Reactome (Pathway browser version 3.7; Reactome database release: 75) with default settings [57]. Proteins with permutation‐based FDR < 0.05 were used for the GO annotation and Reactome pathway analysis. Patient survival was plotted in KM plotter (kmplot.com) with basal breast cancer patients (split patients by autoselect best cut‐off) [58]. The phosphoproteomic data were analysed in the ingenuity pathway analysis (ipa) (QIAGEN, Germantown, MD, USA, version from 2018) for pathway enrichment and perseus for motif enrichment analysis.

All the r codes used for figure plotting in this study could be found on https://github.com/QiongW56/UGDH_Publication_2021.

## 3 Results

### 3.1 The proteomic differences based on cell‐of‐origin outweigh proteomic changes that accompany EMT

Three breast EMT cell models consisting of epithelial and mesenchymal breast cell line pairs were used in this study (Fig. 1), namely, D492/D492M (D492 EMT model), HMLE/HMLEM (HMLE EMT model) and PMC42LA/PMC42ET (PMC42 EMT model). All three epithelial cell lines presented a typical cobblestone‐shaped epithelial cell phenotype, while all the mesenchymal cells showed flattened mesenchymal morphology with undefined cell contour (Fig. S1B). The three EMT cell models presented different luminal/myoepithelial/basal phenotypes, with all three models possessing certain degrees of basal breast cell properties.

**Fig. 2:**
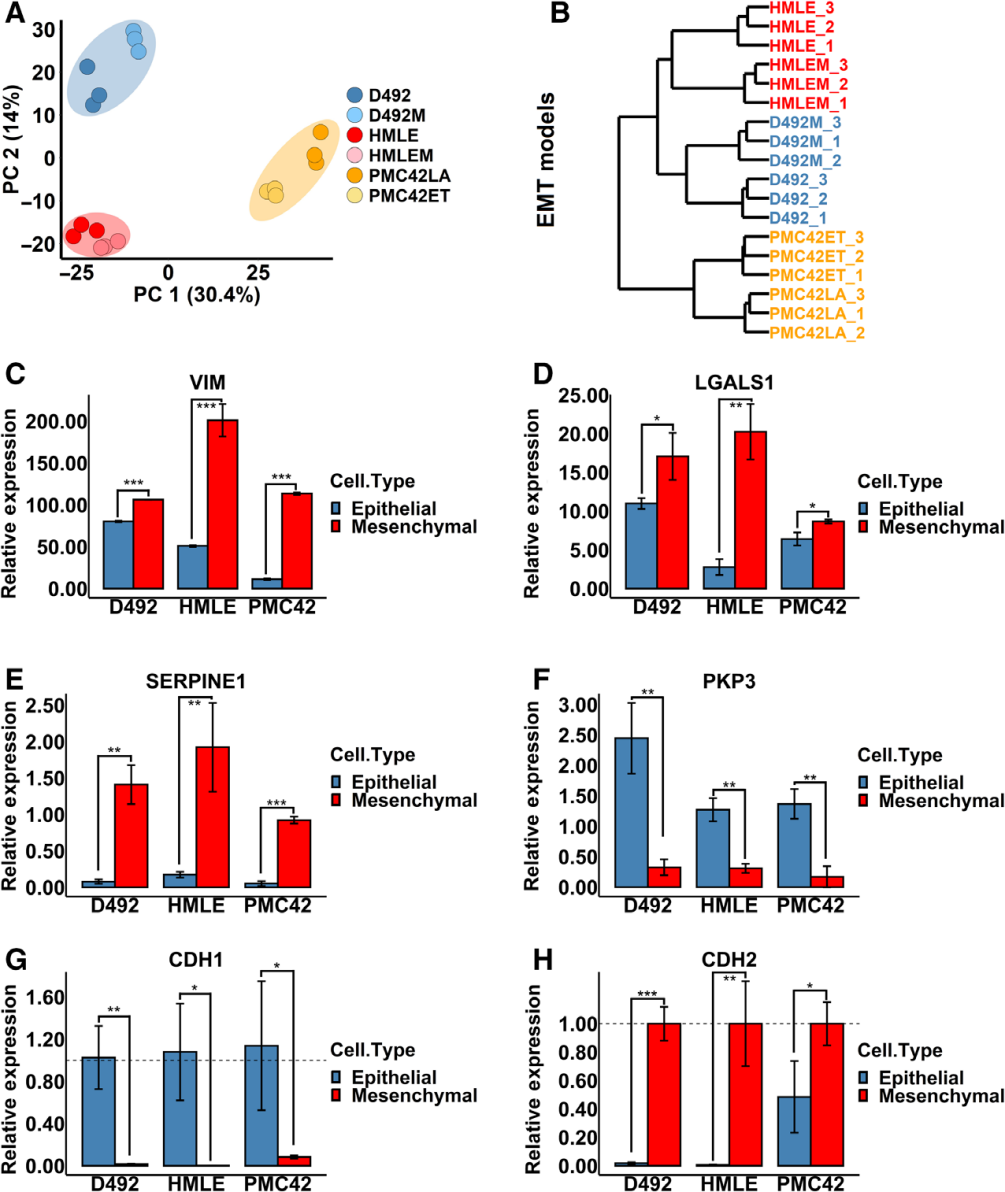
EMT markers in the three breast EMT cell models. (A, B) Proteomic analysis of the three EMT models revealed cell lines with the same origin were more similar than their epithelial or mesenchymal states. The D492 EMT model was more similar to the HMLE model than the PMC42 model. Proteins with valid identification and quantification were included in the analysis (Table S3). Protein levels of the known EMT markers VIM (C), LGALS1 (D), SERPINE1 (E) and PKP3 (F) were consistently altered in all EMT models. RNA expression of CDH1 was downregulated (G), while RNA expression of CDH2 was upregulated after EMT (H). Student's *t*‐test, *P < 0.05; **P < 0.01; ***P < 0.001; *n* = 3. The error bars indicate standard deviation. VIM, vimentin; LGALS1, galectin‐1; SERPINE1, plasminogen activator inhibitor 1; PKP3, plakophilin‐3; CDH1, E‐cadherin; CDH2, N‐cadherin.

Irrespective of being epithelial or mesenchymal, cell lines of the same origin were grouped on the proteomic level (Fig. 2A). The PMC42 model shared the least similarities with the other EMT models (Fig. 2B). To confirm the epithelial and mesenchymal phenotypes on the molecular level, we quantified the EMT markers captured by the proteomic analysis from an EMT marker database [59]. VIM, LGALS1 and SERPINE1 were consistently upregulated in the mesenchymal cells, while PKP3 was downregulated (Fig. 2C–F). Not all EMT markers found in this study were, however, consistently altered among all three models, that is CD44, LMNB1, MSN, FLNA, TLN1, FSCN1, EGFR, S100A2 and NDRG1 (Fig. S2). Since E‐cadherin (CDH1) and N‐cadherin (CDH2), two typical EMT markers [60], were not covered in the proteomic analysis, we checked the expression of these by real‐time PCR. CDH1 was significantly downregulated, while CDH2 was significantly upregulated in all EMT models (Fig. 2G,H).

### 3.2 Cell–cell and cell–extracellular matrix interactions are altered in EMT, and a diversity of pathways and molecular activities are changed in D492 as opposed to protein translation in HMLE and PMC42

Heterogenicity and plasticity are two intrinsic characteristics of EMT. To further define the epithelial and mesenchymal cells in all three EMT models, we compared their proteomes with respect to the number and profile of the significantly altered proteins along with their biological function and identified consistent EMT markers.

**Fig. 3:**
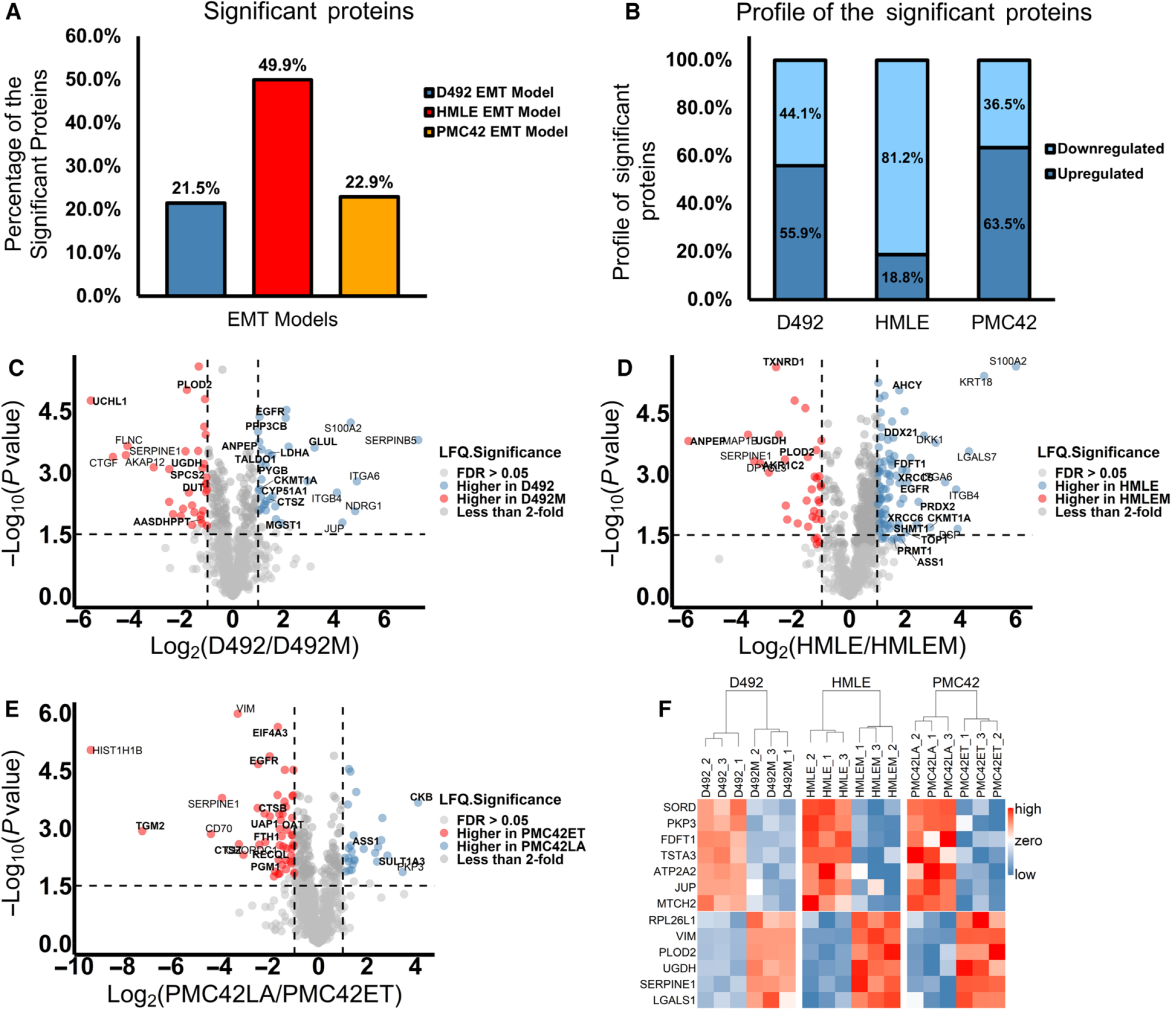
Proteomic analysis of the three breast EMT cell models. (A) Percentages of significantly altered proteins in the EMT models (permutation‐based FDR < 0.05). (B) Up‐ and downregulation profiles for all the significantly changed proteins in the EMT models. The log2(epithelial/mesenchymal ratio) along with the −log10(*P*‐value) for each protein was plotted for the D492 model (C), HMLE model (D) and PMC42 model (E). Proteins with FDR (permutation‐based) less than 0.05 and fold change more than 2 are coloured. The horizontal and vertical dashed lines indicate a *P*‐value of 0.03 [−log10(*P*‐value) = 1.5] and a 2‐fold change, respectively. The annotated proteins had a log2(fold change) of more than 3. Proteins involved in metabolism with a log2(fold change) of more than 1 for D492 model, 1.5 for HMLE and PMC42 models were bold label marked. *n* = 3. (F) A list of proteins significantly changed in the same direction (Student's *t*‐test, *P* < 0.05) in all three EMT models. SERPINE1, RPL26L1, PLOD2, UGDH, LGALS1 and VIM were upregulated, while JUP, PKP3, MTCH2, ATP2A2, FDFT1, SORD and TSTA3 were downregulated after EMT.

In total, 873 proteins were deemed valid proteins in identification and quantification (Table S3). In the D492 model, 188 out of the 873 valid proteins (21.5%) were significantly changed after EMT (permutation‐based FDR < 0.05). In the HMLE model, 436 out of 873 proteins (49.9%) were significantly altered, while 200 proteins (22.9%) were significantly changed in the PMC42 model (Fig. 3A). Out of the significantly altered proteins, 55.9% (105/188) in the D492 model, 18.8% (82/436) in the HMLE model, while 63.5% (127/200) in the PMC42 model were upregulated after EMT (Fig. 3B). To ensure reproducibility of the proteomic data used in this study (Table S4), we compared the current proteomic data set with the previously generated data for the D492 EMT model [26]. The correlation coefficient of these two data sets was 0.936 (Fig. S3).

We next filtered the identified proteins based on their log2 fold changes and −log10 *P*‐values (Fig. 3C–E) and summarized the consistently altered proteins in all three EMT models (Fig. 3F and Table 1) to identify common changes in EMT. A literature search for each EMT target revealed that all had been associated with EMT previously, albeit to a different extent (Table 1). To evaluate these consistently altered EMT markers in the context of cancer progression, we confirmed the expression of these markers in the tumorigenic breast mesenchymal cell line D492HER2. All the targets detected in D492HER2 showed the same trends in changes (Table 1).

**Table 1:** The EMT targets were significantly different (Student's *t*‐test, *P* < 0.05) in all EMT models. The literature related to each target in terms of EMT was also listed. Changes in these targets in another breast mesenchymal cell line with tumorigenicity were consistent with the findings in this study.

UniProt ID	Protein name	Gene name	Log2(D492/D492M)	Log2(HMLE/HMLEM)	Log2(PMC42LA/PMC42ET)	−Log(*P*‐value D492s)	−Log(*P*‐value HMLEs)	−Log(*P*‐value PMC42s)	EMT‐related literature	Gene expression in mesenchymal cells with tumorigenicity (Log2 ratio)
P05121‐1	Plasminogen activator inhibitor 1	*SERPINE1*	−4.236	−3.442	−3.993	3.416	3.313	3.780	[70, 72, 97]	−4.739
Q9UNX3	60S ribosomal protein L26‐like 1	*RPL26L1*	−2.367	−1.123	−1.346	1.981	2.349	1.925	[98]	Not detected
O00469‐1	Procollagen‐lysine, 2‐oxoglutarate 5‐dioxygenase 2	*PLOD2*	−1.817	−1.504	−1.121	5.005	3.411	2.072	[70, 71]	−1.687
O60701	UDP‐glucose 6‐dehydrogenase	*UGDH*	−1.193	−2.550	−0.779	3.101	3.962	2.285	[27, 28, 32, 34, 75]	−0.828
P09382	Galectin‐1	*LGALS1*	−0.623	−2.912	−0.444	1.769	3.035	1.799	[67, 69, 70]	−0.422
P08670	Vimentin	*VIM*	−0.407	−1.979	−3.343	5.493	4.802	5.970	[4]	−0.957
Q13630	GDP‐l‐fucose synthase	*TSTA3*	0.553	1.009	0.508	2.871	2.517	1.789	[99, 100]	0.562
Q00796	Sorbitol dehydrogenase	*SORD*	0.599	1.459	1.452	2.457	2.957	2.806	[101]	0.111
P37268	Squalene synthase	*FDFT1*	0.683	1.536	0.491	3.510	3.215	1.462	[102]	0.722
P16615	Sarcoplasmic/endoplasmic reticulum calcium ATPase 2	*ATP2A2*	0.919	0.523	0.641	2.388	1.551	2.316	[103]	0.299
Q9Y6C9	Mitochondrial carrier homolog 2	*MTCH2*	1.239	0.725	0.899	2.219	1.624	3.015	[99]	0.198
Q9Y446	Plakophilin‐3	*PKP3*	2.958	2.057	3.478	2.786	3.077	1.850	[66, 68]	3.458
P14923	Junction plakoglobin	*JUP*	4.343	1.541	1.214	1.783	1.356	3.611	[104, 105]	4.344

**Fig. 4:**
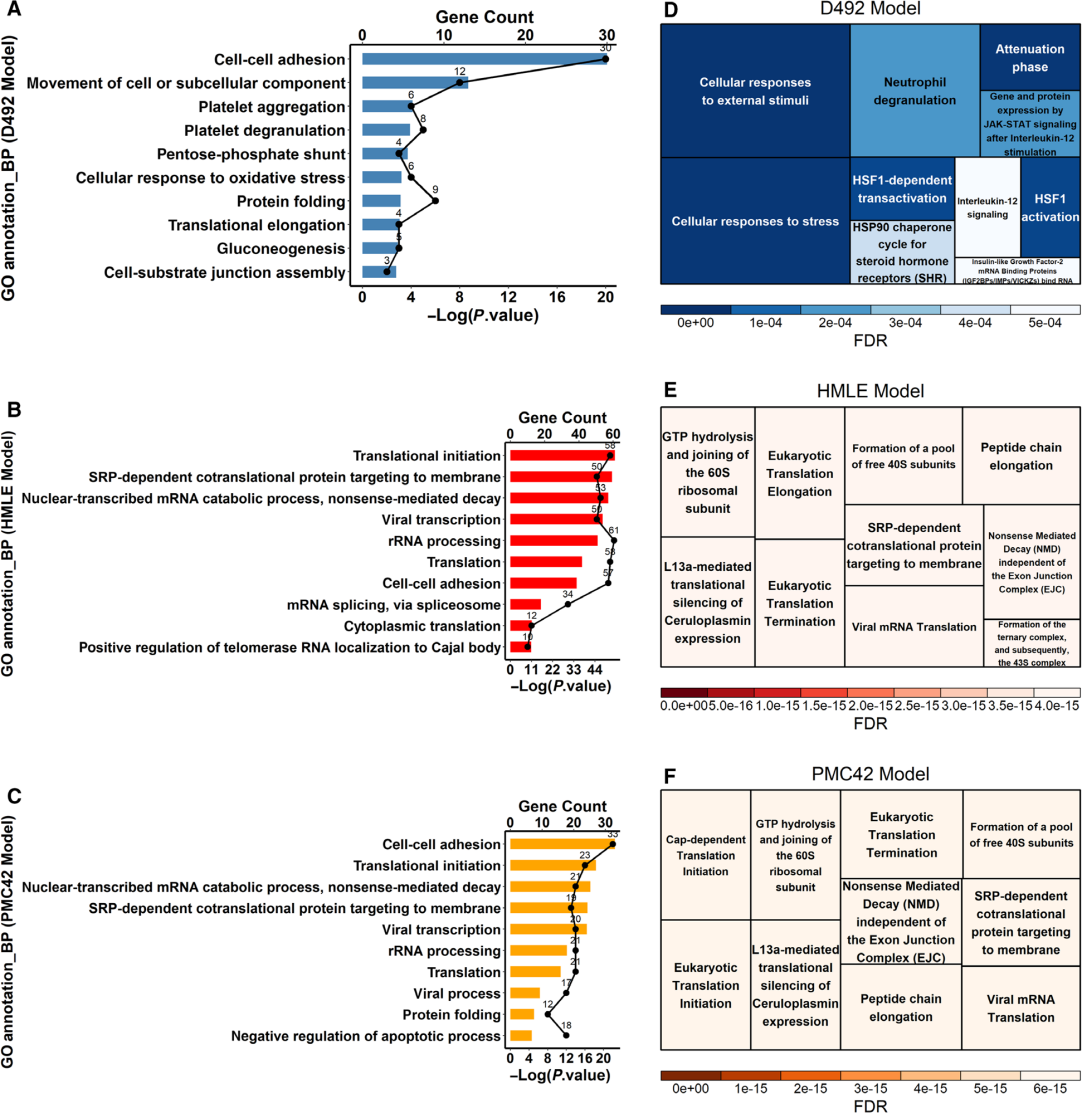
Functional annotation of the GO terms (BP) and Reactome pathway analysis for the three EMT models. (A–C) Functional annotation of the GO terms (BP) was conducted on the DAVID platform (DAVID Bioinformatics Resources 6.8) for the D492 model (A), HMLE model (B), and PMC42 model (C). The GO terms were listed according to the −log10 *P*‐value in descending order. The numbers of genes in each GO term were also plotted as dots/line plots. (D–F) Reactome pathway analysis (Pathway browser version 3.7; Reactome database release: 75) for the D492 model (D), HMLE model (E) and PMC42 model (F). Data used for both the GO annotation and the pathway analysis (Table S3) were proteins significantly different in each EMT model (permutation‐based FDR < 0.05). Default settings in the DAVID and Reactome platforms were used. BP, biological process.

To define functional changes in EMT, we annotated the GO terms for the significantly changed proteins (Table S3) and observed that the Biological Process (BP) ‘cell–cell adhesion’ was altered in all three EMT models (Fig. 4A–C). The D492 model had the least similarity compared with the other two models, with only one common BP term (i.e. cell–cell adhesion) out of the top 10 enriched BP terms (Fig. 4A). In contrast, the PMC42 model shared its top seven terms with HMLE (Fig. 4B,C). The same trend was observed using enriched Reactome pathway analysis (Fig. 4D–F). The altered Reactome pathways in the D492 model were related to response to cell stress, IGF signalling and interleukin‐12 signalling (Fig. 4D). In both the HMLE and PMC42 models, changes were, however, mainly to pathways involved in the protein translational process (Fig. 4E,F). Comparison of changes to cellular components (Fig. S4A–C) and molecular function (Fig. S4D–F) was similarly indicative of more similarities in changes to protein function following EMT in the HMLE and PMC42 models as compared to the D492 model.

### 3.3 UGDH is negatively correlated with patient survival and affects cell proliferation, cell invasion and SNAI1 expression

**Fig. 5:**
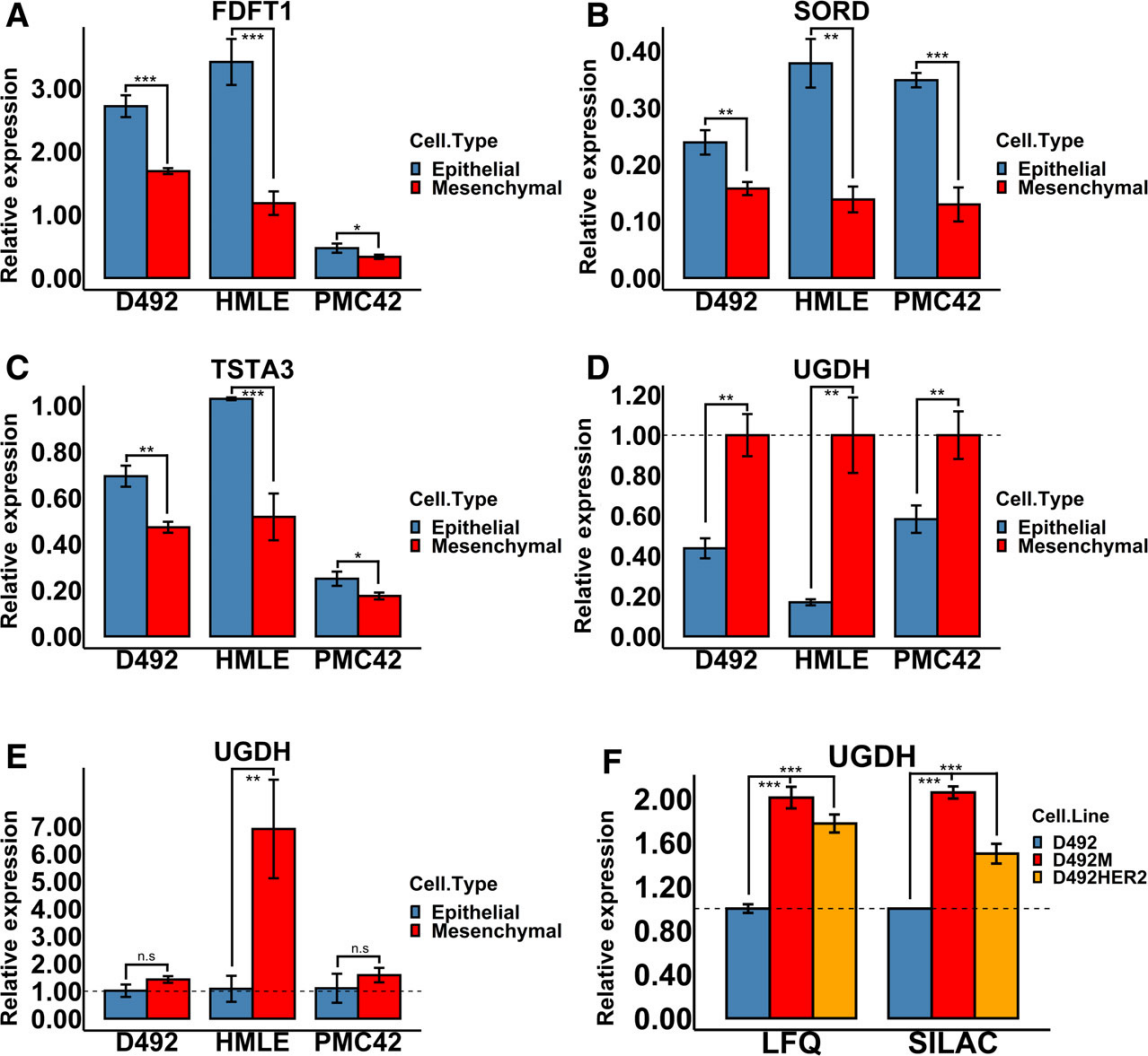
Four metabolic enzymes changed consistently in all EMT models. (A–D) The proteomic analyses revealed that the metabolic enzymes FDFT1, SORD, TSTA3 and UGDH changed consistently in all EMT models. (E) The RNA level of UGDH in all EMT models was consistently higher in the mesenchymal cell lines. (F) The UGDH protein level in the epithelial and mesenchymal cells was confirmed in another data set [26] and further confirmed in another tumorigenic breast mesenchymal cell line D492HER2. Student's *t*‐test, *P < 0.05; **P < 0.01; ***P < 0.001; *n* = 3. The error bars indicate standard deviation. FDFT1, squalene synthase; SORD, sorbitol dehydrogenase; TSTA3, GDP‐l‐fucose synthase; UGDH, UDP‐glucose 6‐dehydrogenase.

Next, we focused on the metabolic changes during EMT. Out of the thirteen identified targets listed in Table 1, four proteins were involved in metabolism: FDFT1, SORD and TSTA3 were downregulated while UGDH was upregulated (Fig. 5A–D). We further tested the RNA expression of UGDH, which showed the most changes to protein expression in all EMT models and was associated with cancer aggressiveness. Though there was no significance in the D492 and PMC42 models, the upregulating trends in all EMT models were seen (Fig. 5E). To relate these findings to breast cancer, we tested the protein level of UGDH in the tumorigenic breast mesenchymal cell line D492HER2. UGDH was upregulated in D492HER2 as observed in nontumorigenic mesenchymal cell line D492M (Fig. 5F).

**Fig. 6:**
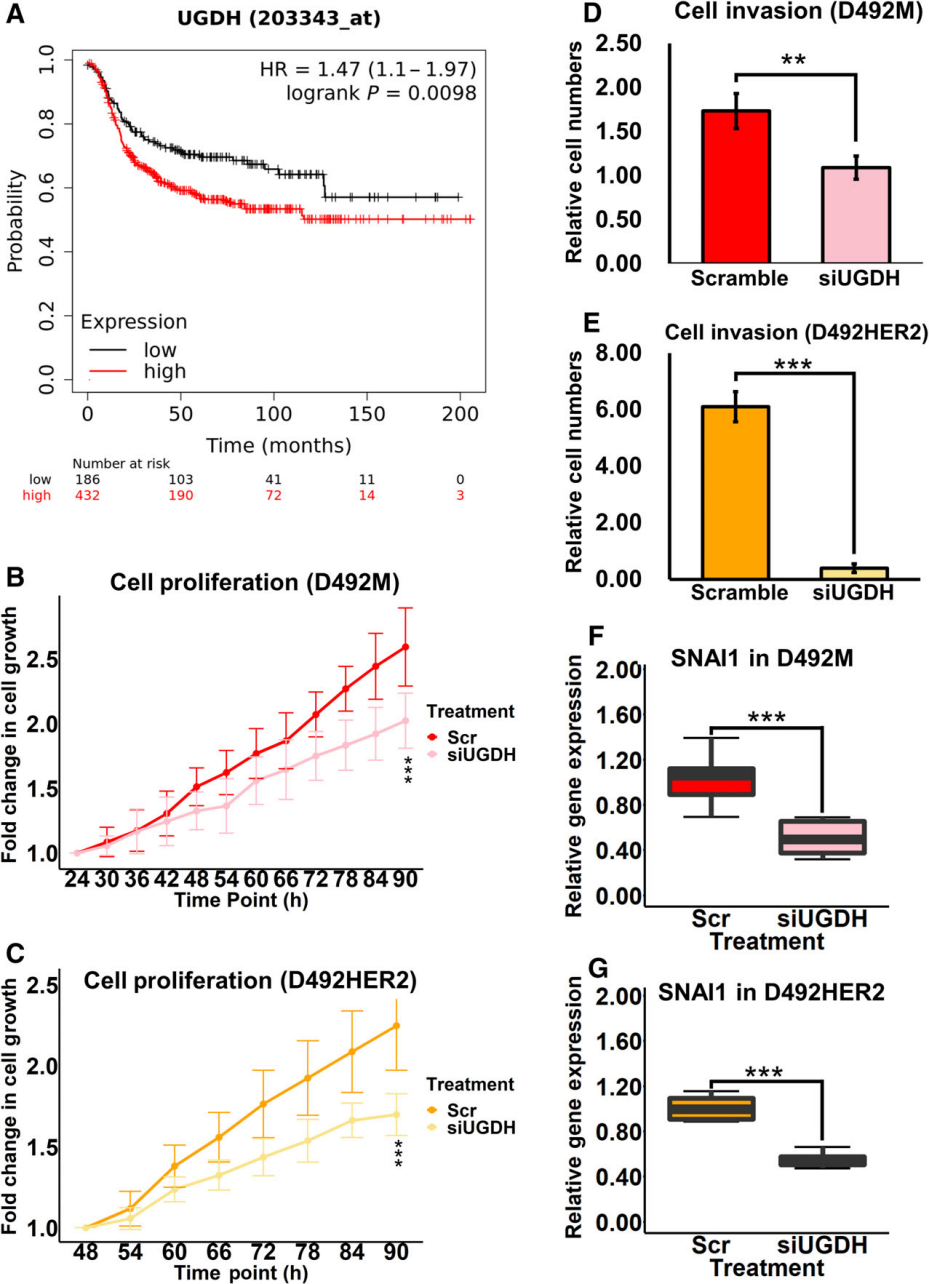
Functional analysis of UGDH in EMT. (A) The Kaplan–Meier plot of UGDH in basal breast cancer patients was downloaded from kmplot.com. (B, C) Cell proliferation slowed down with the siRNA knock‐down of *UGDH* in both nontumorigenic D492M (B) and tumorigenic D492HER2 (C). *n* equals 4, and three spots were chosen for each replicate during the imaging process. (D, E) Cell invasion decreased with *UGDH* knock‐down in both nontumorigenic D492M (D) and tumorigenic D492HER2 (E). *n* equals 3, and 10 spots were chosen for each replicate during the cell counting process. (F, G) One of the main EMT transcription factors SNAI1 was downregulated after the siRNA knock‐down of *UGDH* in both nontumorigenic D492M (*n* = 7) (F) and tumorigenic D492HER2 (*n* = 4) (G). Student's *t*‐test, **P < 0.01; ***P < 0.001. The error bars indicate standard deviation. SNAI1, Snail family transcriptional repressor 1; UGDH, UDP‐glucose 6‐dehydrogenase.

Recent studies have reported that UGDH affects patient survival [34], cell proliferation [32, 37], cell invasion [27], cell migration [34, 37] and SNAI1 expression [28]. We set out to confirm these effects of UGDH in our EMT cell lines. High UGDH level was associated with worse patient survival in basal breast cancer patients based on KM plotter (Fig. 6A). Based on this, we analysed effects of UGDH on cell morphology, proliferation, invasion and SNAI1 expression in two types of breast mesenchymal cells: nontumorigenic D492M and tumorigenic D492HER2 via siRNA‐mediated knock‐down of *UGDH* (Fig. S5). Knock‐down of *UGDH* did not yield observable morphological changes but slowed down cell growth (Fig. 6B,C) and invasion (Fig. 6D,E and Fig. S6A–C) in both cell lines. SNAI1 RNA expression was downregulated after *UGDH* knock‐down, which was also consistent with the literature (Fig. 6F,G and Fig. S6D,E).

### 3.4 GPC is downregulated while NAA is upregulated following *UGDH* knock‐down in the mesenchymal cells

**Fig. 7:**
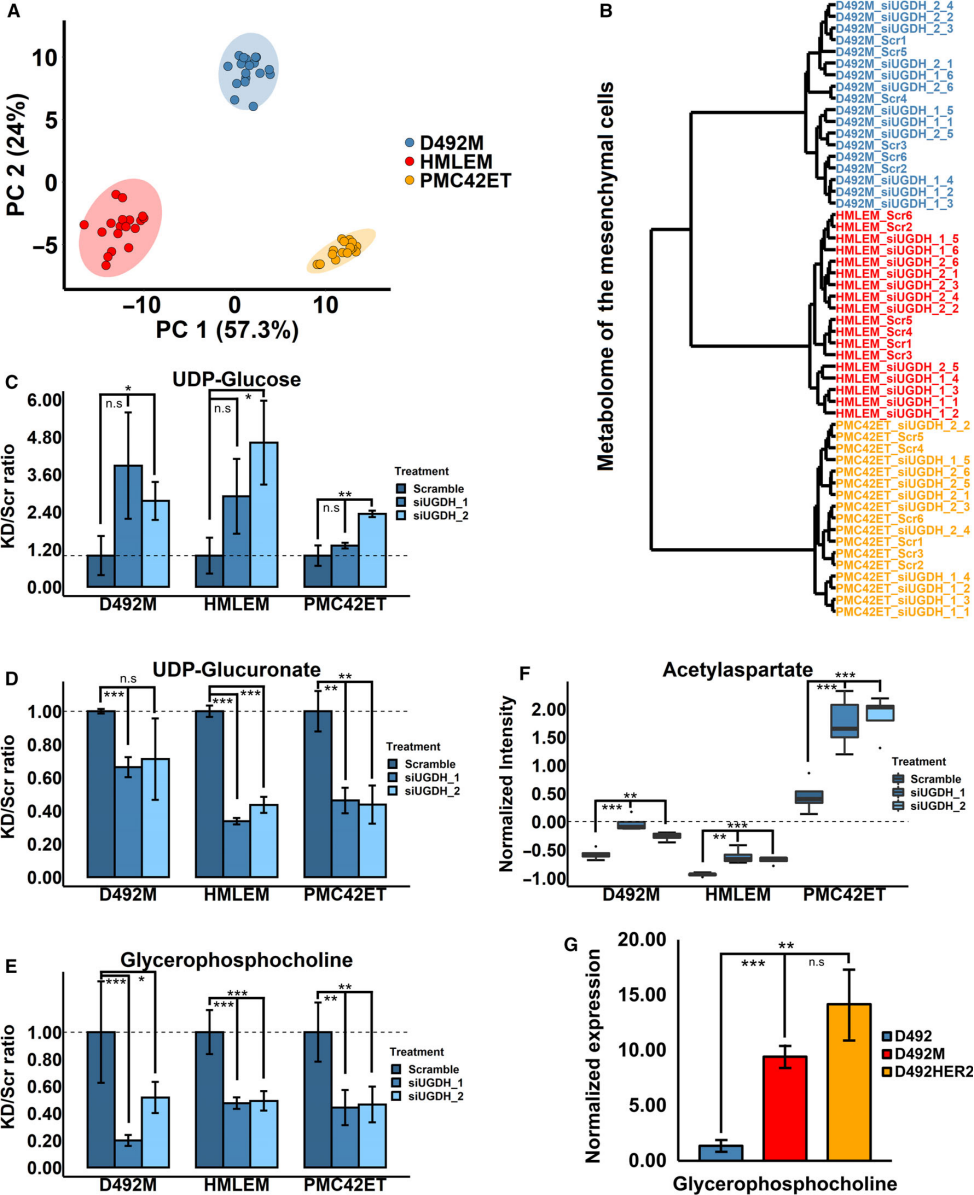
Metabolomic changes after siRNA knock‐down of *UGDH* in the mesenchymal cell lines. (A, B) Metabolomic clustering of the mesenchymal cell lines with different treatments. Valid metabolite identification and quantification from the negative, positive and basic modes were integrated into the analysis. Samples clustered together based on the differences of their metabolome among different EMT models (A), and the D492 mesenchymal cells were closer to the HMLE mesenchymal cells than PMC42 at the metabolic level (B). (C) The UGDH substrate UDP‐glucose was increased following siRNA knock‐down of *UGDH* in all cell lines with two siRNAs (*n* = 3). (D) The UGDH product UDP‐glucuronate was decreased with siRNA knock‐down of *UGDH* in all the cell lines with two siRNAs (*n* = 3). (E, F) siRNA knock‐down of *UGDH* significantly decreased GPC (*n* = 5) and increased acetylaspartate (*n* = 4) in all the cell lines with two siRNA treatments. (G) GPC level was higher in the nontumorigenic D492M than the epithelial D492, and it was the highest in the tumorigenic mesenchymal D492HER2 (*n* = 3). Student's *t*‐test, *P < 0.05; **P < 0.01; ***P < 0.001. The error bars indicate standard deviation. UGDH, UDP‐glucose 6‐dehydrogenase.

UGDH catalyses the conversion of UDP‐Glc to UDP‐GlcA that are constituents of glycosaminoglycans and N‐ and O‐linked glycans [61]. To confirm the metabolic impacts of UGDH in mesenchymal cells, we knocked down *UGDH* with siRNAs and performed metabolomic analysis in all three mesenchymal cell lines. Samples from the same cell line clustered together at the metabolic level despite *UGDH* knock‐down (Fig. 7A,B). As with the proteome, the metabolome of D492M was closer to that of HMLEM than the metabolome of PMC42ET. Knock‐down of *UGDH* did not confer a distinct metabolic phenotype compared with the scramble control in any of the mesenchymal cell lines (Fig. 7B). An increasing trend of UDP‐Glc was observed in all the mesenchymal cell lines with all the siUGDH treatments, although nonsignificant for one of the siRNAs (Fig. 7C). UDP‐GlcA decreased in all the mesenchymal cell lines in all the siUGDH treatments, although nonsignificantly with one siRNA in D492M (Fig. 7D).

To better evaluate the systemic changes of UGDH on metabolism, we carried out an untargeted metabolomics analysis. Knocking down *UGDH* significantly decreased the intracellular glycerophosphocholine (GPC) level and increased acetylaspartate (NAA) in all the mesenchymal cell lines (Fig. 7E,F), which was confirmed in the aggressive D492HER2 and MDA‐MB‐231 cell lines (Fig. S7A–D). To investigate whether GPC and NAA were associated with the UGDH level and differently expressed regardless of tumorigenicity, we tested the GPC and NAA levels in the epithelial D492, nontumorigenic mesenchymal D492M and tumorigenic mesenchymal D492HER2 cells. GPC was higher in both D492M and D492HER2 compared with D492 (Fig. 7G). We further looked into the connection between GPC and the mesenchymal state based on published data sets in the literature [62, 63] but did not observe any significant correlation (Fig. S7E and Table S5). siRNA‐mediated knock‐down of *UGDH* did not yield significant and consistent changes to choline and phosphocholine (Fig. S7F,G).

Glycerophosphocholine is part of the choline synthetic pathway from phosphatidylcholine (PtdCho), and NAA is closely associated with acetyl‐CoA and central carbon metabolism. To query how changes to UDP‐GlcA might relate to GPC and NAA processing via changes in metabolic flux, we performed *in silico* knock‐down of *UGDH* using tailored genome‐scale metabolic models of D492 [23, 25]. Changes to metabolic flux were observed within keratan metabolism, hyaluronan processing, pentose phosphate pathway and the central carbon metabolic pathways (Table S6). Negligible changes were, however, observed to GPC production and consumption.

### 3.5 PDGFRB signalling regulates UGDH potentially via NFkB‐p65

**Fig. 8:**
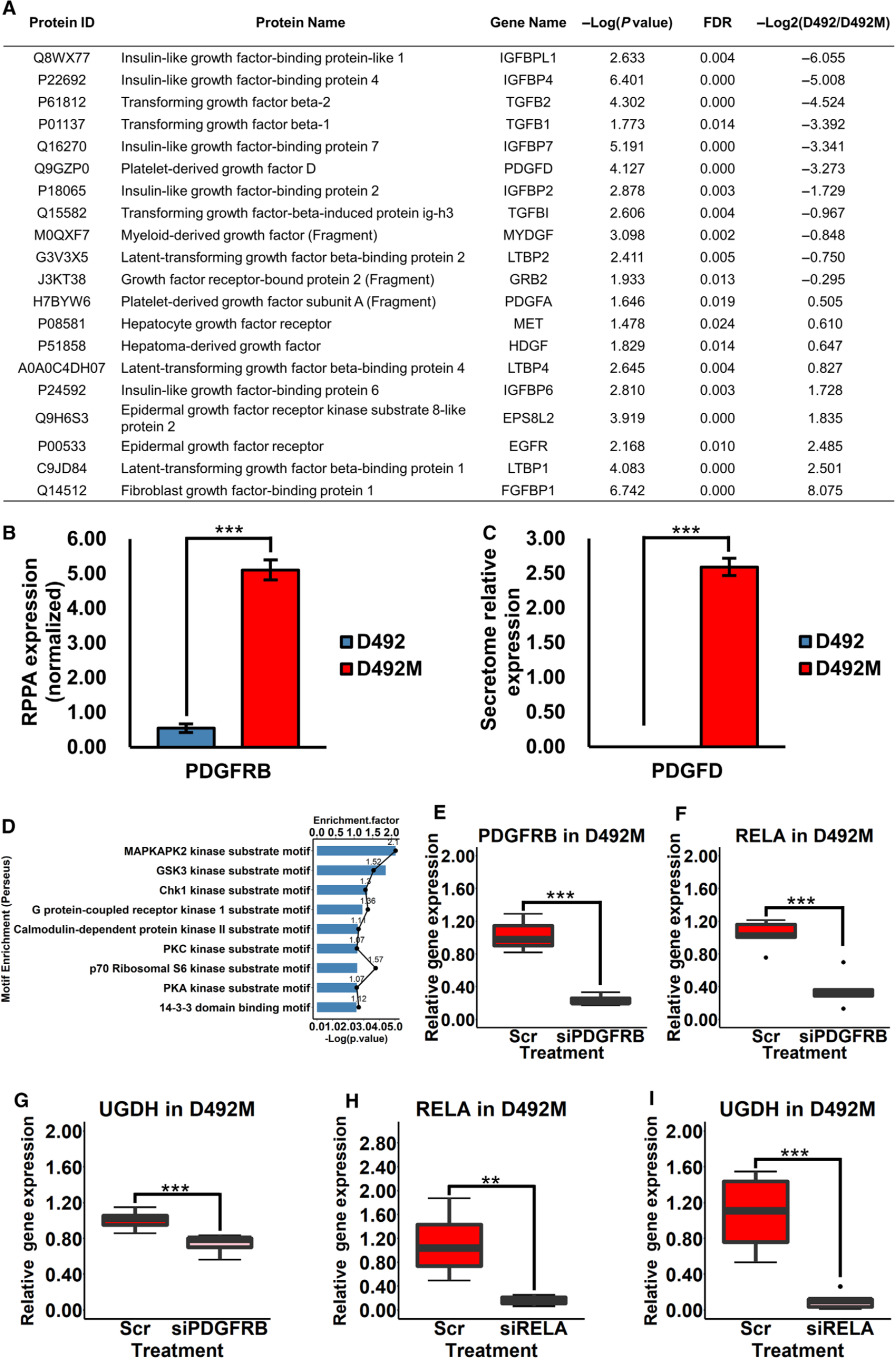
PDGFRB regulates UGDH via RELA (NFkB‐p65) in D492M. (A) Top differently secreted growth factors from the secretome of the D492 EMT model were reported (permutation‐based FDR < 0.05). (B) PDGFRB was highly expressed in mesenchymal cells than epithelial cells in the D492 model on the protein level based on the RPPA analysis (*n* = 3) [65]. (C) PDGFD protein was highly secreted in mesenchymal cells than epithelial cells in the D492 model (*n* = 3). (D) Motif enrichment of the phospho‐proteome in the D492 EMT model suggested that PKC kinase activity, among others, was highly enriched in EMT. Enrichment factors ≥ 2; motif enrichment terms were ranked based on the –log10(*P*‐value). (E) The knock‐down efficiency of *PDGFRB* with siRNA in the D492M cell line was around 80% (*n* = 7). (F) RELA (NFkB‐p65) was downregulated after the siRNA knock‐down of *PDGFRB* in D492M (*n* = 5). (G) UGDH was downregulated after the siRNA knock‐down of *PDGFRB* in D492M (*n* = 7). (H) The knock‐down efficiency of *RELA* with the first siRNA in D492M was about 80% (*n* = 6). (I) UGDH was downregulated after the siRNA knock‐down of *RELA* in D492M with the first siRNA (*n* = 6). Student's *t*‐test, **P < 0.01; ***P < 0.001. The error bars indicate standard deviation. PDGFRB, platelet‐derived growth factor receptor beta; RELA (NFκB‐p65), nuclear factor NF‐kappa‐B p65 subunit; UGDH, UDP‐glucose 6‐dehydrogenase.

We next investigated the upstream regulation of UGDH by analysing the secretome of the D492 model [64]. IGF, TGF‐β and PDGFD signalling regulators were highly presented in the culture medium of D492M cells (Fig. 8A). PDGFRB was highly expressed in the nontumorigenic D492M (Fig. 8B) and the tumorigenic D492HER2 mesenchymal cell lines (Fig. S8A) [65], and PDGFD was secreted by D492M (Fig. 8C). We thus focused on the role of PDGF signalling in UGDH regulation. In addition, the motif enrichment analysis of the phosphorylation sites within the phosphoproteomic data (Table S7) revealed potentially altered kinases in the D492 EMT model, including the downstream target of the PDGF signalling PKC kinase (Fig. 8D). siRNA‐mediated knock‐down of *PDGFRB* decreased both the PDGFR signalling downstream regulator RELA (NFkB‐p65) and UGDH in D492M (Fig. 8E–G) and D492HER2 (Fig. S8B–D). We further investigated the impact of RELA on UGDH and found that siRNA‐mediated knock‐down of *RELA* decreased the UGDH RNA level in D492M (Fig. 8H,I and Fig. S8E,F). We observed the same effect of *RELA* knock‐down on UGDH in D492HER2 with only one siRNA (Fig. S8G–J).

## 4 Discussion

Herein we set out to determine common metabolic changes in cell models used to study EMT in breast epithelium. We chose the D492, HMLE and PMC42 EMT cell models on account of the spontaneous EMT induction approaches and the nontumorigenic properties of these cell lines to ensure the intrinsic characteristics and plasticity of EMT (Fig. 1). First, we validated and compared the EMT cell models on the proteomic level (Fig. 2A,B). Cell lines clustered based on their origin instead of their epithelial or mesenchymal characteristics, indicating that the spontaneous epithelial–mesenchymal switches during EMT/MET are subtle compared with the imprinted intrinsic genetic differences among these cell models.

VIM, LGALS1, SERPINE1, PKP3 and the CDH1‐CDH2 switch were consistently altered in all the EMT models (Fig. 2C–H) and have all been related to EMT in different cancer types [4, 60, 66, 67, 68, 69, 70, 71, 72]. Vimentin, a type III intermediate filament and well‐known EMT marker, shapes the cell structure and modifies cell movements and cell adhesion [73]. SERPINE1, a key player in endothelial homeostasis, is highly upregulated in EMT. However, the function of SERPINE 1 in EMT is poorly understood. The possible role of SERPINE1 in EMT is to affect the function of urokinase‐type plasminogen activator receptor (uPAR) to regulate extracellular matrix degradation [72]. LGALS1 is a carbohydrate‐binding protein. One study shows that upregulation of LGALS1 decreases CDH1 and increases SNAI1 [67]. PKP3 is an epithelial marker and is under the control of the EMT transcriptional regulator ZEB1 [66, 68]. All these EMT markers were consistently altered in the three EMT models (Fig. 2C–F). However, inconsistencies in EMT markers were also observed indicative of their different roles in EMT with respect to cell type. The PMC42 model was different from the other EMT cell models (Fig. S2), potentially reflecting the cell heterogeneity and partially expressed mesenchymal marker CDH2 in the epithelial cells (Fig. 2H) [74]. The consistently altered EMT markers were also confirmed in the tumorigenic breast mesenchymal cell line D492HER2 (Fig. 3F and Table 1), indicating that these makers are not only crucial for EMT but also potentially involved in tumorigenicity and malignancy, even though they are not critical for tumour initiation. Moreover, many of the consistently altered proteins identified in this study remain unexplored in the context of EMT (Table 1).

Our findings confirmed that changes to cellular morphology, cell–cell communication and cell–extracellular matrix interaction are among the main characteristics of EMT (Fig. 4 and Fig. S4). Even though the proteomes of the D492 cell lines were closer to HMLE (Fig. 2B), they shared the least similarity in the altered pathways post‐EMT. The changed translational activities in HMLE and PMC42 and the altered responses to stress and signalling regulation in D492 suggest that in HMLE and PMC42, the epithelial or mesenchymal switch may largely be mediated by altered expression of proteins involved, whereas in D492, post‐translational control of existing proteins may play a more important role. This may also reflect the more stem‐like properties of the D492 epithelial cells that confer cell flexibility. Our findings indicate that distinct and dominant cell properties (e.g. stem cell properties) outweigh similar genetic backgrounds for EMT induction, while cells with disparate genetic backgrounds can rely on similar machineries to induce EMT.

Recently, a growing number of studies have focused on UGDH in cancer, and the roles of UGDH in tumour growth, metastasis and patient survival have been well documented [27, 32, 34, 35, 36, 37]. Additionally, UGDH has been connected to EMT [27, 28, 32, 34, 75]. Arnold et al. [27] reported that UGDH was highly expressed in mesenchymal cells and mesenchymal‐like breast cancers and connected UDP‐GlcA (the enzymatic product of UGDH) to extracellular matrix remodelling and mesenchymal‐like properties. Furthermore, UGDH regulates SNAI1, a well‐known EMT transcription factor, via UDP‐Glc (the enzymatic substrate of UGDH) [28]. We confirmed the upregulation of UGDH in both nontumorigenic and tumorigenic mesenchymal cell lines, suggesting UGDH is associated with the mesenchymal feature in tumorigenic cell lines (Fig. 5F). Interestingly, even though the high expression of UGDH was associated with worse survival in basal breast cancer patients (Fig. 6A) and decreased UGDH jeopardized cell proliferation (Fig. 6B,C) and invasion (Fig. 6D,E and Fig. S6A–C), all the mesenchymal cells in this study possess upregulated UGDH and are nontumorigenic. Thus, elevated UGDH expression is likely not a trigger for tumour initiation, but tumorigenic cells may rely on UGDH to facilitate tumorigenicity and malignancy. UGDH may induce resistance to chemotherapy via drug elimination. This was supported by a recent study demonstrating that high levels of UGDH are correlated with worse prognosis in triple‐negative breast cancer patients receiving chemotherapy, likely by promoting UDP‐GlcA‐mediated detoxification and elimination of epirubicin [76]. The effect of UGDH on SNAI1 supports that UGDH has a regulatory role in EMT and that its function may exceed its catalytic role, perhaps via nonconventional signalling regulatory effects such as glycosylation (Fig. 6F,G and Fig. S6D,E).

The D492 EMT model metabolome was more similar to HMLE than PMC42 (Fig. 7A,B), consistent with the proteomic analysis (Fig. 2A,B). In agreement with the literature, knock‐down of *UGDH* increased UDP‐Glc and decreased UDP‐GlcA (Fig. 7C,D), both of which are important metabolites with wide impact on cells [27, 28]. The most prominently altered metabolite was, however, GPC (Fig. 7E). Increased GPC in tumours indicates changes to choline metabolism, which has emerged as a hallmark of cancer progression [77]. GPC is negatively correlated with patient survival [78] and is high in basal‐like breast cancer xenograft and oestrogen receptor‐negative breast cancer patients [79, 80]. Reduced GPC levels after chemotherapies are associated with better survival in breast cancer patients [78]. D492 and D492M are basal‐like breast cell lines, while D492HER2, deemed as HER2‐positive breast cell line, is more closely associated with the aggressive claudin low than other breast cancer types [26, 81]. Claudin low is not a distinct intrinsic breast tumour subtype but may permeate various breast cancer types including HER2‐positive [82]. The higher levels of GPC along with UGDH in basal‐like mesenchymal D492M and claudin‐low D492HER2 are in congruence with the clinical observations. GPC may be involved in EMT, but the connection between GPC and EMT in cancer is unclear [83, 84]. Li and colleagues detected GPC in 928 cell lines and performed different types of metabolite‐gene association analyses. They reported various genes associated with GPC where the EMT master regulator TWIST1 was one of the top hits [62]. The insignificant correlation between GPC and mesenchymal cells (Fig. S7E) suggests that the increased GPC levels observed in the D492 mesenchymal cells are results of one or several regulators independent of mesenchymal traits. Our results support that GPC in part is regulated by the mesenchymal metabolic enzyme UGDH, but the molecular mechanisms underlying this warrant further investigation. *In silico* knock‐down of *UGDH* in the genome‐scale metabolic models revealed several metabolic changes (Table S6) primarily on account of rerouting of glucose flux away from UDP‐GlcA formation and into glycolysis and associated pathways (e.g. PPP and TCA), which may be partially responsible for the increased NAA (Fig. 7F). It, however, imparted no changes in GPC, implying that the changes to metabolic fluxes encircling GPC due to a mass‐action effect of UGDH are likely secondary to changes that arise through altered glycosylation. Cell osmotic pressure balance is vital for normal cell functions and cell survival. GPC is a well‐known intracellular osmotic regulator, and proteoglycans serve as extracellular osmolytes [85]. The decreased intracellular GPC may thus counterbalance the decreased extracellular osmotic pressure induced by the reduced proteoglycans caused by the knock‐down of *UGDH*.

Recently, studies have shown that UGDH regulates signalling factors and lipid metabolic genes, such as SNAI1‐, SIP‐1‐, ERK/MAPK‐, SIX1‐ and PPARγ‐targeted genes [27, 28, 32, 34]. PPARγ is a nuclear transcription factor regulating genes linked to lipid metabolism [86, 87] that interacts with choline/PtdCho metabolism [88]. UGDH has been proposed to inhibit PPAR signalling and affect lipid metabolism [27]. Consistent with this, we observed a negative association between UGDH expression and PPARγ signalling (Fig. S7H), suggesting the *UGDH* knock‐down decreases intracellular GPC level via PPARγ. Moreover, phospholipase A2 group XV (PLA2G15), which belongs to Cytosolic phospholipase A2 (cPLA2), is an enzyme catalysing the hydrolysis of phospholipids, potentially involved in the formation of GPC from PtdCho, and is under the control of ERK signalling [89, 90]. Knock‐down of *UGDH* has been reported to downregulate the phosphorylation of ERK (pERK) in highly invasive ovarian cancer cells [32]. We observed that both GPC and PLA2G15 were higher in the mesenchymal cell lines D492M and D492HER2 (Fig. 7G and Fig. S7I), implying UGDH may regulate GPC via pERK‐PLA2G15. Taken together, UGDH may indirectly affect GPC via signalling regulations and/or lipid metabolism to retain the osmotic balance across the cell membrane, although further investigations are needed. Furthermore, the absence of UGDH in the list of genes associated with GPC reported in the literature indicates UGDH may not be a dominant GPC regulator [62].

Slit2, SP1, TGF‐β, hypoxia, p38^MAPK^, LMP2A and PI3K/Akt affect and/or regulate UGDH expression, which highlights that UGDH is under complex regulation network control [29, 30, 31, 33]. These regulators are potentially mediated by PDGF signalling that, along with the downstream transcription factor NFkB, is dysregulated in cancer progression and EMT [7, 91, 92]. Tam et al. [7] reported a switch from EGFR to PDGFR signalling in cancer stem cell formation and EMT. The higher expression of PDGFRB and secretion of PDGFD in D492M compared with D492 suggest PDGFRB signalling is upregulated in mesenchymal cells (Fig. 8A–C and Fig. S8A) supported by the increased phospholipase C, PI3K/Akt and PKCα signalling (Fig. S7H and Fig. 8D) since these are well‐known downstream targets of PDGFR [7, 93]. This is consistent with PDGFD‐PDGFRB signal regulation of EMT [94, 95]. NFkB‐p65 is a downstream regulator of PDGFR signalling [92, 96]. Downregulating either *PDGFRB* or *NFkB‐p65* decreased UGDH expression on the RNA level in both D492M and D492HER2. However, the impacts of PDGFRB and NFkB‐p65 on UGDH were dampened in D492HER2 compared with D492M (Fig. 8E–I and Fig. S8B–J). We have previously noticed that D492HER2 is a less complete mesenchymal cell line than D492M, suggesting that the regulations of PDGFRB and NFkB‐p65 on UGDH are more dominant in complete mesenchymal cells [26]. It thus appears that UGDH is part of an interactive signalling and metabolic network in which PDGFRB differently regulates UGDH via NFkB‐p65 depending on specific cell types.

## 5 Conclusions

**Fig. 9:**
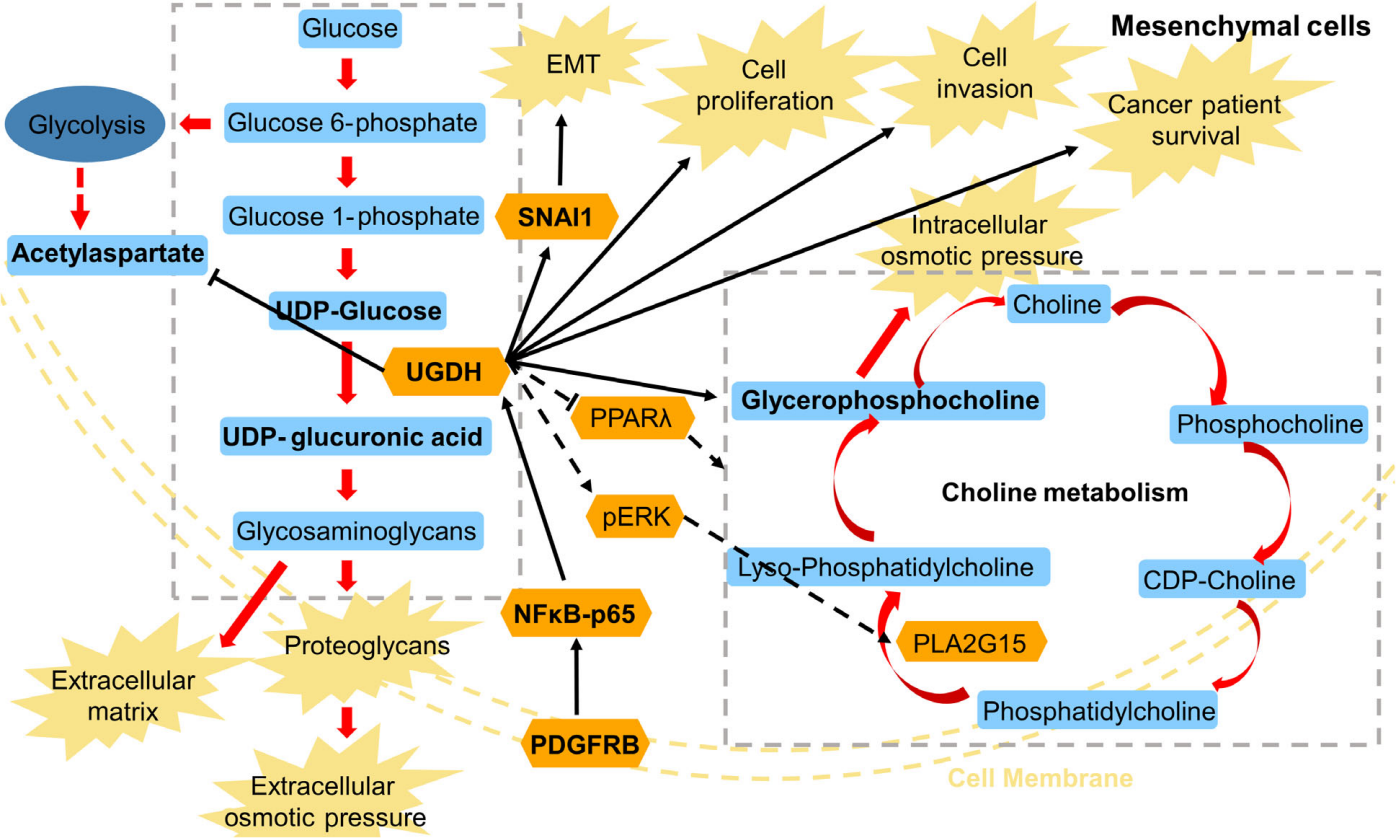
Summary of the study. This figure illustrates the main metabolic pathways and findings involved in this study. UGDH catalyses UDP‐glucose into UDP‐glucuronic acid, an indispensable unit for glycosaminoglycans, proteoglycans and extracellular matrix. In this study, UGDH was found to be highly expressed in the mesenchymal cells and affect cancer patient survival, mesenchymal cell proliferation and invasion and the EMT transcription factor SNAI1, and it was under the control of the PDGFRB‐NFκB pathway. In addition, the knock‐down of *UGDH* with siRNAs significantly decreased the intracellular GPC levels and increased the acetylaspartate (NAA) levels in all the mesenchymal cell lines. NAA is closely linked to the central carbon metabolism and potentially affected by the mass‐action effects of *UGDH* knock‐down. GPC is an intracellular osmolyte and part of the choline metabolism. Knock‐down of *UGDH* hindered the formation of proteoglycans and further decreased the extracellular osmotic pressure, which could be counteracted by the reduced intracellular osmotic pressure induced by GPC. Based on the literature, we hypothesized that to ease the osmotic stress, knock‐down of *UGDH* affected PPARλ‐lipid metabolism and/or pERK‐PLA2G15 to regulate GPC. However, further studies are needed to address this question. UGDH, UDP‐glucose 6‐dehydrogenase; SNAI1, Snail family transcriptional repressor 1; PDGFRB, platelet‐derived growth factor receptor beta; RELA (NFκB‐p65), nuclear factor NF‐kappa‐B p65 subunit; PPARγ, peroxisome proliferator‐activated receptor γ; pERK, phosphorylated extracellular signal‐regulated kinase; PLA2G15, phospholipase A2 group XV.

In conclusion, we used three breast EMT cell models to study proteomic changes in EMT, focusing on metabolic reprogramming. We further studied the downstream functions of the metabolic enzyme UGDH in cancer progression and metabolism, and finally, we explored the upstream signalling regulating UGDH (Fig. 9). Several proteins were found to be involved in the EMT programme and likely to participate in normal human breast gland development, that is SERPINE1, RPL26L1, PLOD2, UGDH, LGALS1, VIM, TSTA3, SORD, FDFT1, ATP2A2, MTCH2, PKP3 and JUP, within which, UGDH, TSTA3, SORD and FDFT1 were metabolic enzymes with UGDH possessing the biggest difference between the epithelial and mesenchymal cell lines. UGDH regulated SNAI1, affected cell proliferation and invasion and is associated with patient survival potentially via regulation of the intracellular GPC level. PDGFRB was involved in the regulation of UGDH in mesenchymal cells, likely through NFkB‐p65. Further studies on understanding the roles of UGDH on GPC and its relationship with EMT could be valuable in developing novel therapeutics against breast cancer.

## Conflict of interest

The authors declare no conflict of interest.

## Author contributions

QW conceived the study, carried out the experiments, performed the data analysis, designed the figures and wrote the manuscript. STK performed the metabolomic untargeted analysis and the flux analysis in the genome‐scale metabolic network reconstructions (GEMs); FJ conducted the metabolomic experiments; AIV carried out experiments; LH, DMF and AS performed the proteomic analysis under the supervision of GS. OR conceived, supervised and funded the study, as well as analysed the data and wrote the manuscript. All authors provided critical feedbacks on the manuscript and data analysis.

### Peer Review

The peer review history for this article is available at https://publons.com/publon/10.1002/1878‐0261.13172.

## Supporting information


**Fig. S1.** Study workflow and the phenotypes of the cell lines. (A) Workflow of the proteomic analysis of the three breast EMT cell models and metabolomics analysis after siRNA knock‐down of the metabolic target *UGDH* in all the mesenchymal cell lines. Three breast EMT cell models (epithelial and mesenchymal cell line pairs) were used in this study, D492&D492M, HMLE&HMLEM, and PMC42LA&PMC42ET. The proteomic strategy was label‐free quantification (LFQ) with each cell line in triplicates. The metabolomic strategy was untargeted metabolomics in negative, positive, and basic modes with six replicates. The upstream signaling regulation and downstream cellular functions of UGDH were also investigated in this study. The tumorigenic breast mesenchymal cell line D492HER2 and malignant MDA‐MB‐231 were employed further to define the functions of UGDH in tumor malignancy. (B) Photos of all the cell lines in the three breast EMT cell models used in this study were shown. Different cell lines were cultured in their routine maintaining medium respectively, and the photos were taken under phase contrast with objectives 5x or 20x.

**Fig. S2.** Inconsistent EMT markers. A list of known EMT markers (based on the public EMT database dbEMT) was inconsistently altered among the three EMT models. Student's T‐test, *: *P* < 0.05; **: *P* < 0.01; ***: *P* < 0.001; n = 3. CD44, CD44 antigen; LMNB1, Lamin‐B1; MSN, Moesin; FLNA, Filamin‐A; TLN1, Talin‐1; FSCN1, Fascin; EGFR, Epidermal growth factor receptor; S100A2, S100 calcium binding protein A2; NDRG1, N‐myc downstream regulated 1.

**Fig. S3.** Accuracy and validity of the proteomic analysis. The accuracy and validity of the proteomic analysis in this study were confirmed by comparing the current data to our previously generated proteomic data for the D492 EMT model [26]. The correlation between these two datasets was 0.936. The high correlation coefficient (Pearson correlation, 0.936) of the datasets ensures good accuracy and validity of the proteomic analysis in this study. It laid the foundation for valid conclusions deducted from this study.

**Fig. S4.** Functional annotation of the GO terms (CC and MF) for the three EMT models. Functional annotation of the GO terms (CC and MF) was conducted on the DAVID (DAVID Bioinformatics Resources 6.8) platform for each EMT model. Data used for the GO annotation analysis (Supplementary Table 3) were proteins significantly altered in each EMT model (Permutation‐based FDR < 0.05). Default settings were used for the analysis. The GO terms were listed according to the ‐log10 p value in descending order. The numbers of genes in each GO term were also plotted as dots/line plots. CC: Cellular Component; MF: Molecular Function.

**Fig. S5.** Knock‐down efficiency of *UGDH* with two siRNAs. (A‐D) The knock‐down efficiency of *UGDH* with two siRNAs compared to the scramble control was around 80 % in D492M (n = 7 for the first siRNA; n = 9 for the second siRNA) (A‐B) and 60 % in D492HER2 (n = 5) (C‐D). (E) The knock‐down efficiency of *UGDH* with two siRNAs in the metabolomics experiments for D492M, HMELM, and PMC42ET was 90 % (n = 5). KD: Knock‐down. Student's T‐test, ***: *P* < 0.001. UGDH, UDP‐glucose 6‐dehydrogenase.

**Fig. S6.** Functional analysis of UGDH in EMT. (A) Photos of the D492M and D492HER2 cells following knock‐down of *UGDH* via two siRNAs in the invasion assay. Cells were stained with DAPI and observed under the objective 10x. (B‐C) Cell invasion decreased with the second siRNA knock‐down of *UGDH* in both non‐tumorigenic D492M (B) and tumorigenic D492HER2 (C). n equals 3, and ten spots were chosen for each replicate during the cell counting process. (D‐E) One of the main EMT transcription factors SNAI1 was downregulated following the second siRNA knock‐down of *UGDH* in both non‐tumorigenic D492M (n = 5) (D) and tumorigenic D492HER2 (n = 4) (E). (F‐H) The Kaplan‐Meier plots of FDFT1, SORD, and TSTA3 in basal breast cancer patients were downloaded from kmplot.com. Student's T‐test, **: *P* < 0.01; ***: *P* < 0.001. UGDH, UDP‐glucose 6‐dehydrogenase; SNAI1, Snail Family Transcriptional Repressor 1; FDFT1, Squalene synthase; SORD, Sorbitol dehydrogenase; TSTA3, GDP‐L‐fucose synthase.

**Fig. S7.** GPC and NAA were altered with the siUGDH treatment in D492HER2 and MDA‐MB‐231. (A‐D) The glycerophosphocholine (GPC) level was decreased, and the acetylaspartate (NAA) level was increased after the siUGDH treatment in the tumorigenic D492HER2 (n = 3) and malignant MDA‐MB‐231 (n = 6) cell lines. (E) There were no significant differences to the GPC levels between mesenchymal cells and non‐mesenchymal cells based on published datasets in literature [62, 63] (Supplementary Table 5). (F‐G) The expression levels of choline (F) and phosphocholine (G) with *UGDH* knock‐down in the three EMT cell models. No significant and consistent changes were observed for both metabolites in all the cell lines (n = 5). (H) UGDH has been reported to downregulate PPARγ [27]. To test if there was a negative correlation between UGDH and PPAR signaling, we performed a phosphoproteomic analysis on the D492 EMT cell model (Supplementary Table 7) and noticed that the PPAR signaling was downregulated in the mesenchymal cells where UGDH was highly expressed. The IPA pathways were listed based on the –log10(p value), and the z‐scores for the pathways were represented by the dots/line plot. Red: higher in the mesenchymal D492M; blue: higher in the epithelial D492. (I) The enzyme PLA2G15 potentially involved in the hydrolysis of phosphatidylcholine (PtdCho) into GPC was higher in both D492M and D492HER2. UGDH has been reported to regulate the phosphorylation of ERK (pERK) [32]. cPLA2 is responsible for GPC synthesis from PtdCho in choline metabolism and is under the control of ERK/MAPK [89, 90]. We also observed that PLA2G15 was highly expressed in D492M and D492HER2 compared to D492 (I), suggesting the knock‐down of *UGDH* may downregulate GPC via pERK‐PLA2G15 (n = 3). Student's T‐test, *: *P* < 0.05; **: *P* < 0.01; ***: *P* < 0.001. n.s: not significant. UGDH, UDP‐glucose 6‐dehydrogenase; PLA2G15, Phospholipase A2 group XV.

**Fig. S8.** PDGFRB regulates UGDH via RELA (NFkB‐p65). (A) PDGFRB was highly expressed in the tumorigenic mesenchymal cell line D492HER2 based on the RPPA analysis (n = 3) [65]. (B) The knock‐down efficiency of *PDGFRB* with siRNA in the D492HER2 cell line was about 90 % (n = 6). (C) RELA (NFkB‐p65) was downregulated after the siRNA knock‐down of *PDGFRB* in D492HER2 (n = 6). (D) UGDH was downregulated after the siRNA knock‐down of *PDGFRB* in D492HER2 (n = 6). (E) The knock‐down efficiency of *RELA* with the second siRNA in D492M was around 70 % (n = 6). (F) UGDH was downregulated after the knock‐down of *RELA* in D492M with the second siRNA (n = 6). (G) The knock‐down efficiency of *RELA* with the first siRNA in D492HER2 was around 90 % (n = 6). (H) No significant change in UGDH was observed after the knock‐down of *RELA* with the first siRNA in D492HER2 (n = 6). (I) The knock‐down efficiency of *RELA* with the second siRNA in the D492HER2 cell line was about 90 % (n = 6). (J) UGDH was downregulated after the knock‐down of *RELA* in D492HER2 with the second siRNA (n = 6). Student's T‐test, *: *P* < 0.05; **: *P* < 0.01; ***: *P* < 0.001. UGDH, UDP‐glucose 6‐dehydrogenase; PDGFRB, Platelet‐derived growth factor receptor beta; RELA (NFκB‐p65), Nuclear factor NF‐kappa‐B p65 subunit.

**Table S1.** The internal standard mix used in the metabolomics analysis.
**Table S2.** A list of primers used in this study.

**Table S3.** Perseus output data.

**Table S4.** Raw data of proteomics.

**Table S5.** Publicly available data on the GPC levels of mesenchymal cells.
**Table S6.** *In silico* knockdown of UGDH in GEMs.

**Table S7.** Data of phosphoproteomics.

## Data Availability

The mass spectrometry proteomic data have been deposited to the ProteomeXchange Consortium via the PRIDE [44] partner repository with the data set identifier PXD024164. The mass spectrometry phosphoproteomic data have been deposited to the ProteomeXchange Consortium via the PRIDE [44] partner repository with the data set identifier PXD025858.
