# Supplementary figures and images for: UDP‐glucose dehydrogenase expression is upregulated following EMT and differentially affects intracellular glycerophosphocholine and acetylaspartate levels in breast mesenchymal cell lines
Source: Mol Oncol. 2022 Feb 3;16(9):1816–40. doi: 10.1002/1878-0261.13172 (PMC9067156; doi:10.1002/1878-0261.13172)

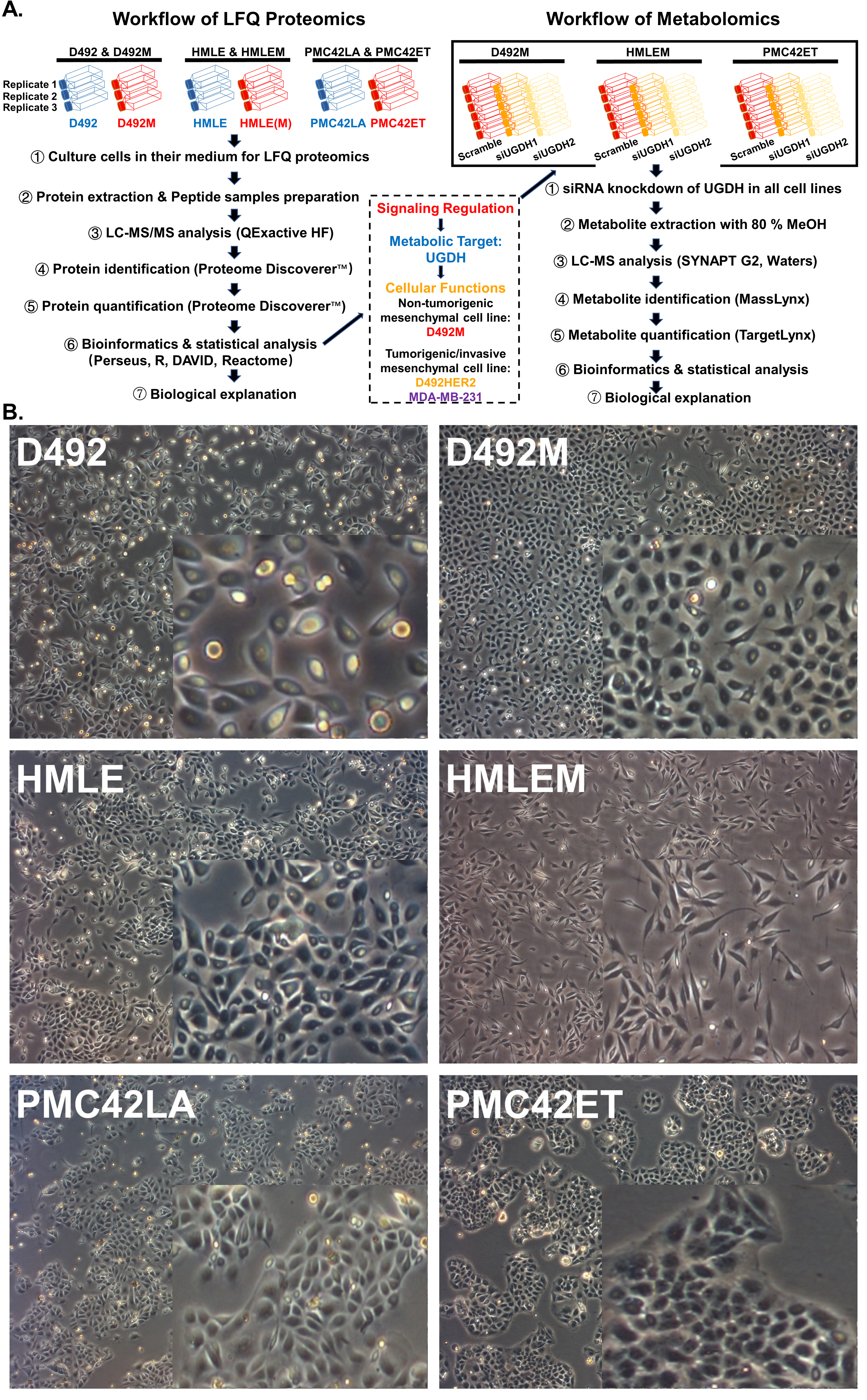

Supplement: Supplementary file 1 — Fig. S1. Study workflow and the phenotypes of the cell lines. (A) Workflow of the proteomic analysis of the three breast EMT cell models and metabolomics analysis after siRNA knock‐down of the metabolic target UGDH in all the mesenchymal cell lines. Three breast EMT cell models (epithelial and mesenchymal cell line pairs) were used in this study, D492&D492M, HMLE&HMLEM, and PMC42LA&PMC42ET. The proteomic strategy was label‐free quantification (LFQ) with each cell line in triplicates. The metabolomic strategy was untargeted metabolomics in negative, positive, and basic modes with six replicates. The upstream signaling regulation and downstream cellular functions of UGDH were also investigated in this study. The tumorigenic breast mesenchymal cell line D492HER2 and malignant MDA‐MB‐231 were employed further to define the functions of UGDH in tumor malignancy. (B) Photos of all the cell lines in the three breast EMT cell models used in this study were shown. Different cell lines were cultured in their routine maintaining medium respectively, and the photos were taken under phase contrast with objectives 5x or 20x. [file MOL2-16-1816-s008.tif]

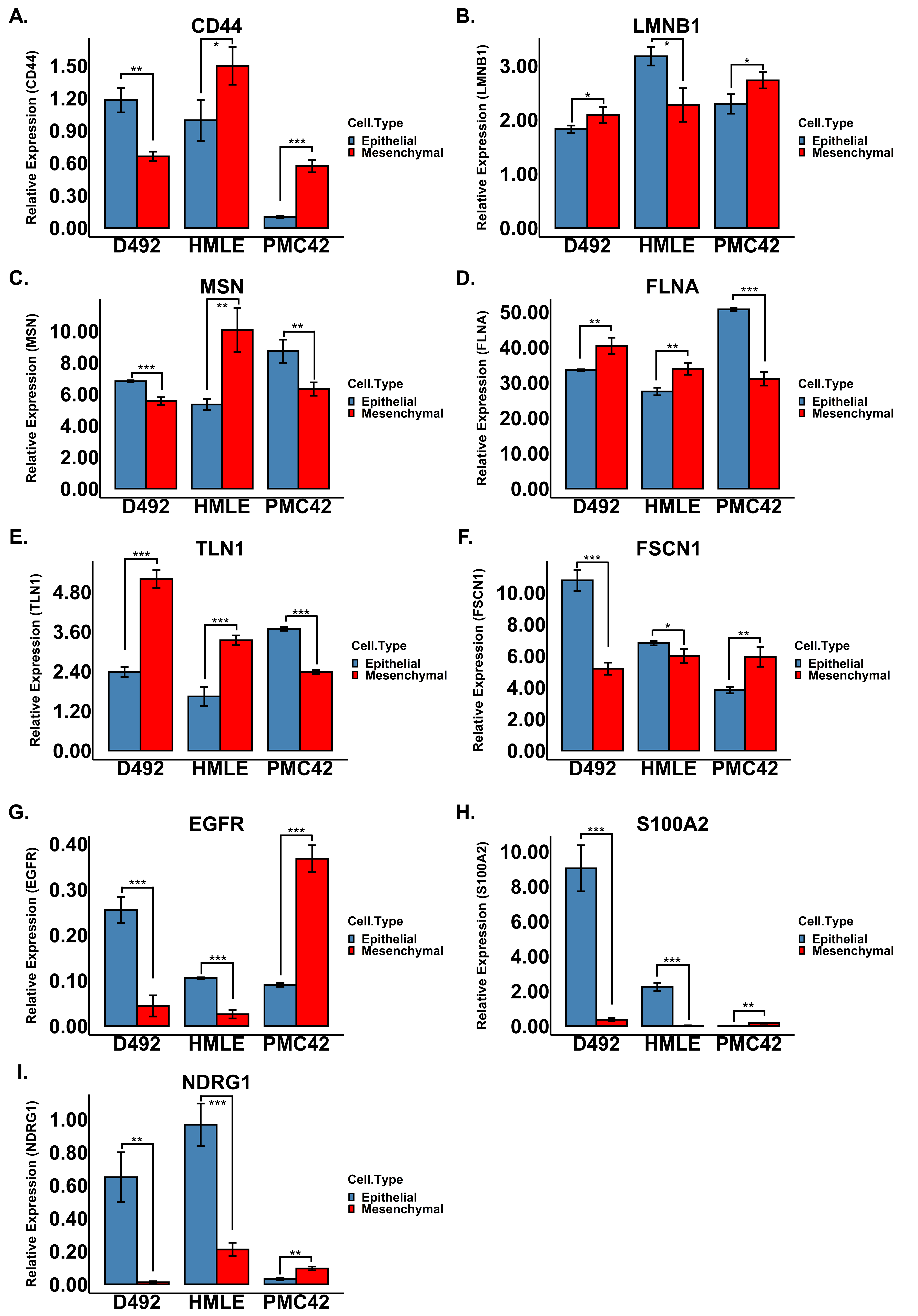

Supplement: Supplementary file 2 — Fig. S2. Inconsistent EMT markers. A list of known EMT markers (based on the public EMT database dbEMT) was inconsistently altered among the three EMT models. Student's T‐test, *: P < 0.05; **: P < 0.01; ***: P < 0.001; n = 3. CD44, CD44 antigen; LMNB1, Lamin‐B1; MSN, Moesin; FLNA, Filamin‐A; TLN1, Talin‐1; FSCN1, Fascin; EGFR, Epidermal growth factor receptor; S100A2, S100 calcium binding protein A2; NDRG1, N‐myc downstream regulated 1. [file MOL2-16-1816-s006.tif]

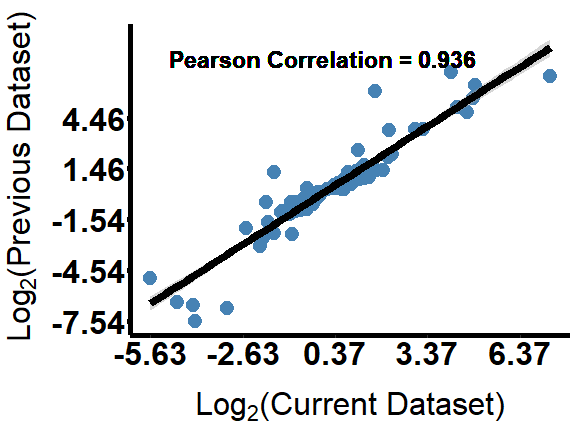

Supplement: Supplementary file 3 — Fig. S3. Accuracy and validity of the proteomic analysis. The accuracy and validity of the proteomic analysis in this study were confirmed by comparing the current data to our previously generated proteomic data for the D492 EMT model [26]. The correlation between these two datasets was 0.936. The high correlation coefficient (Pearson correlation, 0.936) of the datasets ensures good accuracy and validity of the proteomic analysis in this study. It laid the foundation for valid conclusions deducted from this study. [file MOL2-16-1816-s010.tiff]

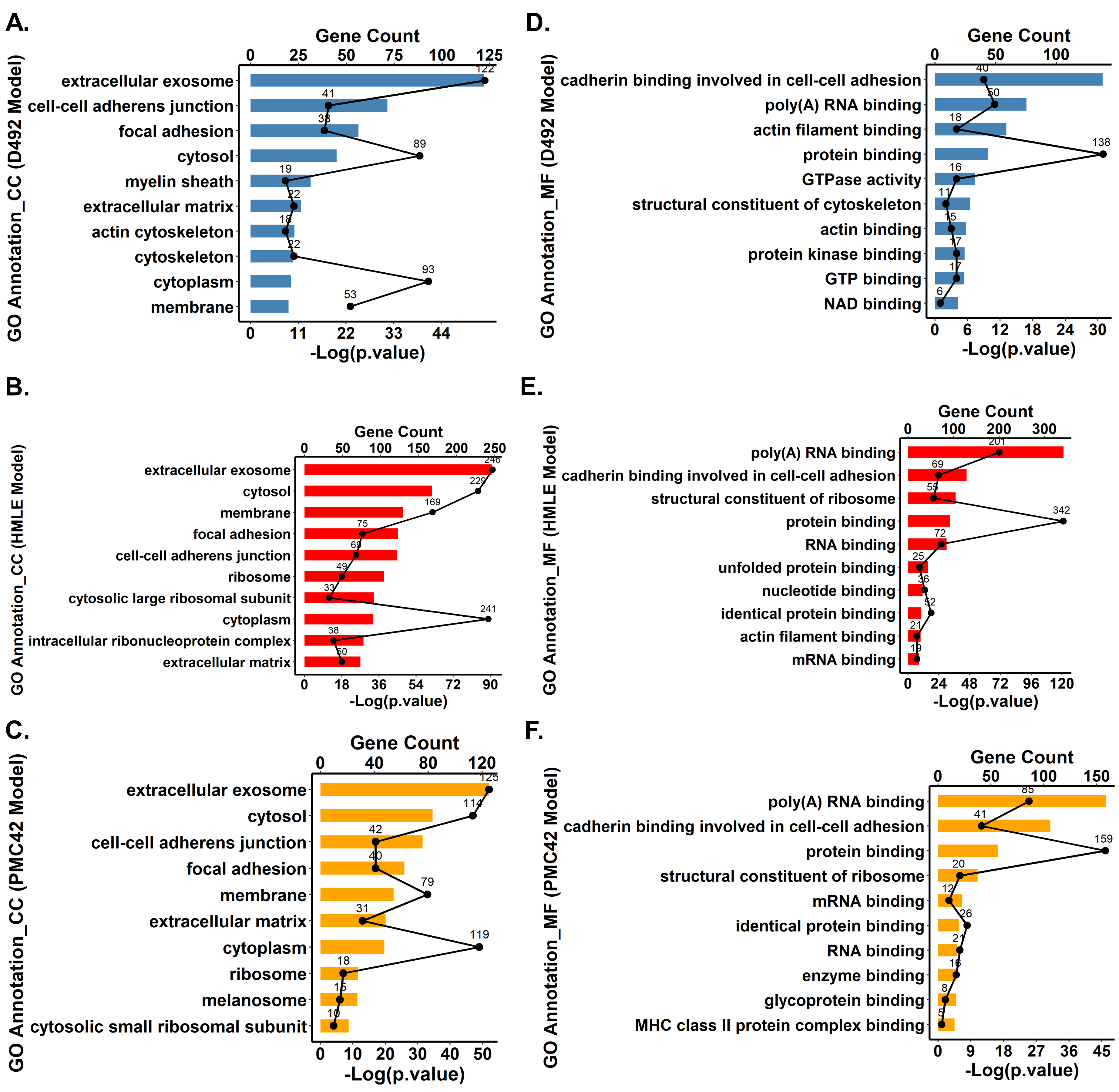

Supplement: Supplementary file 4 — Fig. S4. Functional annotation of the GO terms (CC and MF) for the three EMT models. Functional annotation of the GO terms (CC and MF) was conducted on the DAVID (DAVID Bioinformatics Resources 6.8) platform for each EMT model. Data used for the GO annotation analysis (Supplementary Table 3) were proteins significantly altered in each EMT model (Permutation‐based FDR < 0.05). Default settings were used for the analysis. The GO terms were listed according to the ‐log10 p value in descending order. The numbers of genes in each GO term were also plotted as dots/line plots. CC: Cellular Component; MF: Molecular Function. [file MOL2-16-1816-s004.tif]

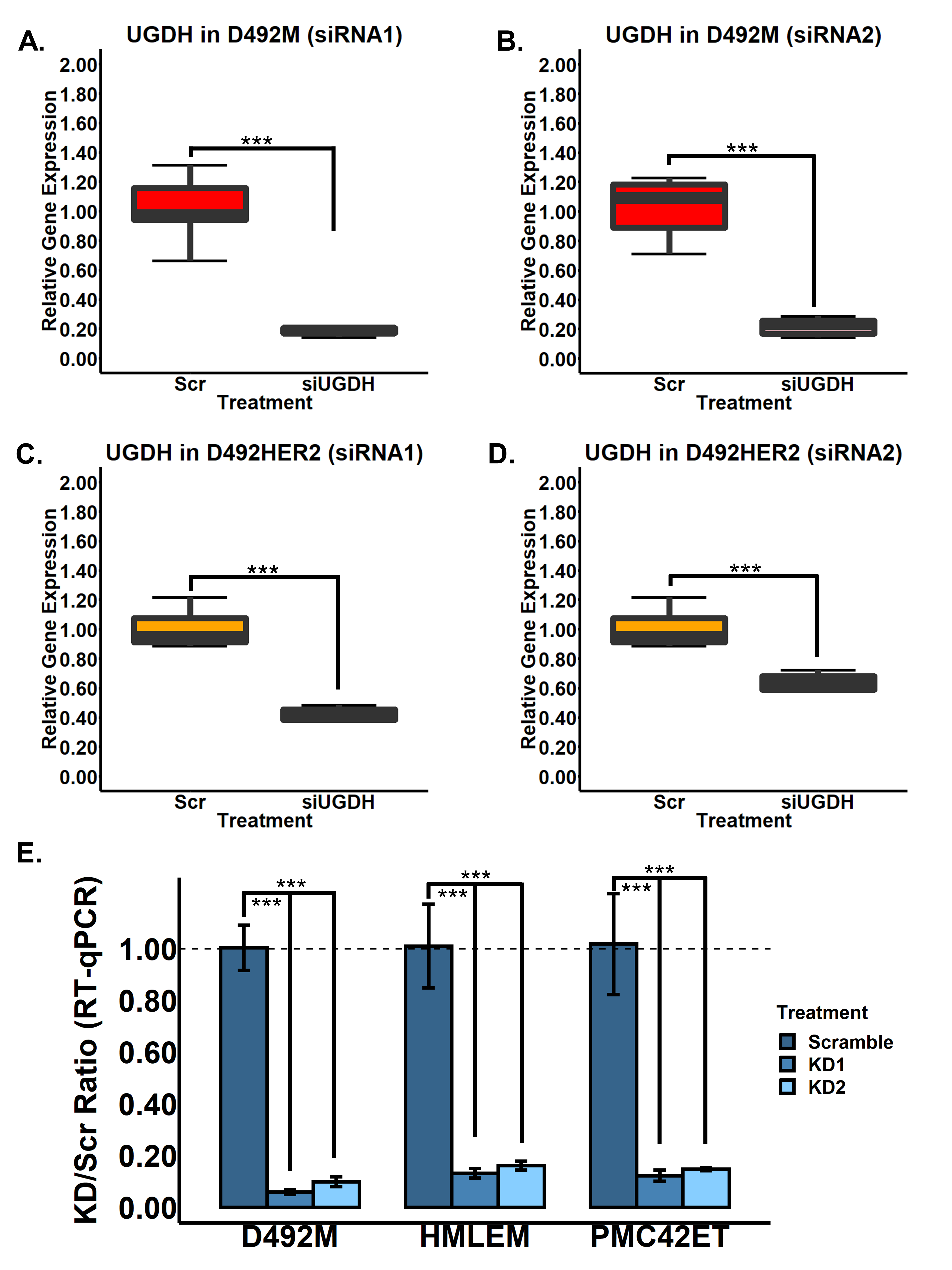

Supplement: Supplementary file 5 — Fig. S5. Knock‐down efficiency of UGDH with two siRNAs. (A‐D) The knock‐down efficiency of UGDH with two siRNAs compared to the scramble control was around 80 % in D492M (n = 7 for the first siRNA; n = 9 for the second siRNA) (A‐B) and 60 % in D492HER2 (n = 5) (C‐D). (E) The knock‐down efficiency of UGDH with two siRNAs in the metabolomics experiments for D492M, HMELM, and PMC42ET was 90 % (n = 5). KD: Knock‐down. Student's T‐test, ***: P < 0.001. UGDH, UDP‐glucose 6‐dehydrogenase. [file MOL2-16-1816-s005.tif]

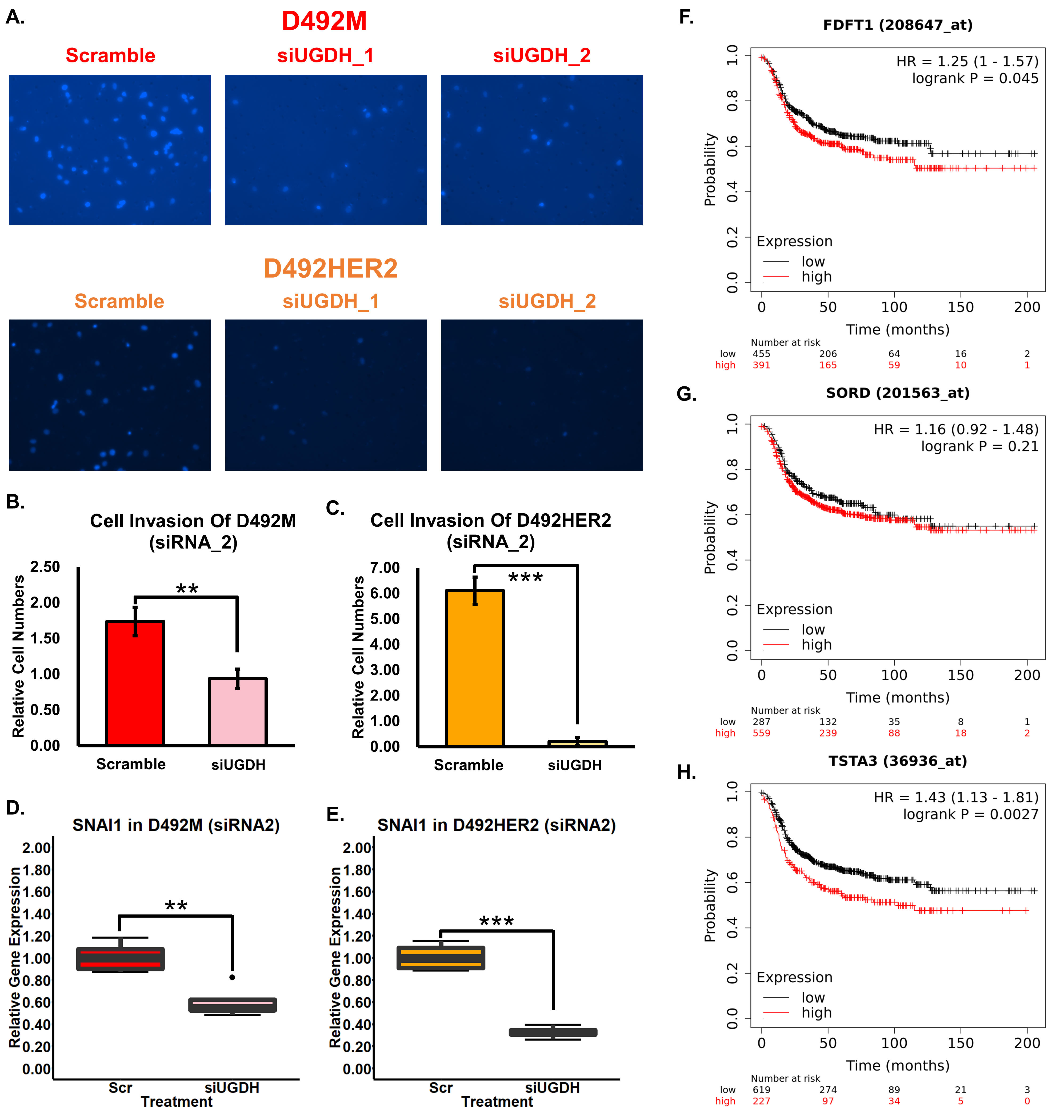

Supplement: Supplementary file 6 — Fig. S6. Functional analysis of UGDH in EMT. (A) Photos of the D492M and D492HER2 cells following knock‐down of UGDH via two siRNAs in the invasion assay. Cells were stained with DAPI and observed under the objective 10x. (B‐C) Cell invasion decreased with the second siRNA knock‐down of UGDH in both non‐tumorigenic D492M (B) and tumorigenic D492HER2 (C). n equals 3, and ten spots were chosen for each replicate during the cell counting process. (D‐E) One of the main EMT transcription factors SNAI1 was downregulated following the second siRNA knock‐down of UGDH in both non‐tumorigenic D492M (n = 5) (D) and tumorigenic D492HER2 (n = 4) (E). (F‐H) The Kaplan‐Meier plots of FDFT1, SORD, and TSTA3 in basal breast cancer patients were downloaded from kmplot.com. Student's T‐test, **: P < 0.01; ***: P < 0.001. UGDH, UDP‐glucose 6‐dehydrogenase; SNAI1, Snail Family Transcriptional Repressor 1; FDFT1, Squalene synthase; SORD, Sorbitol dehydrogenase; TSTA3, GDP‐L‐fucose synthase. [file MOL2-16-1816-s003.tif]

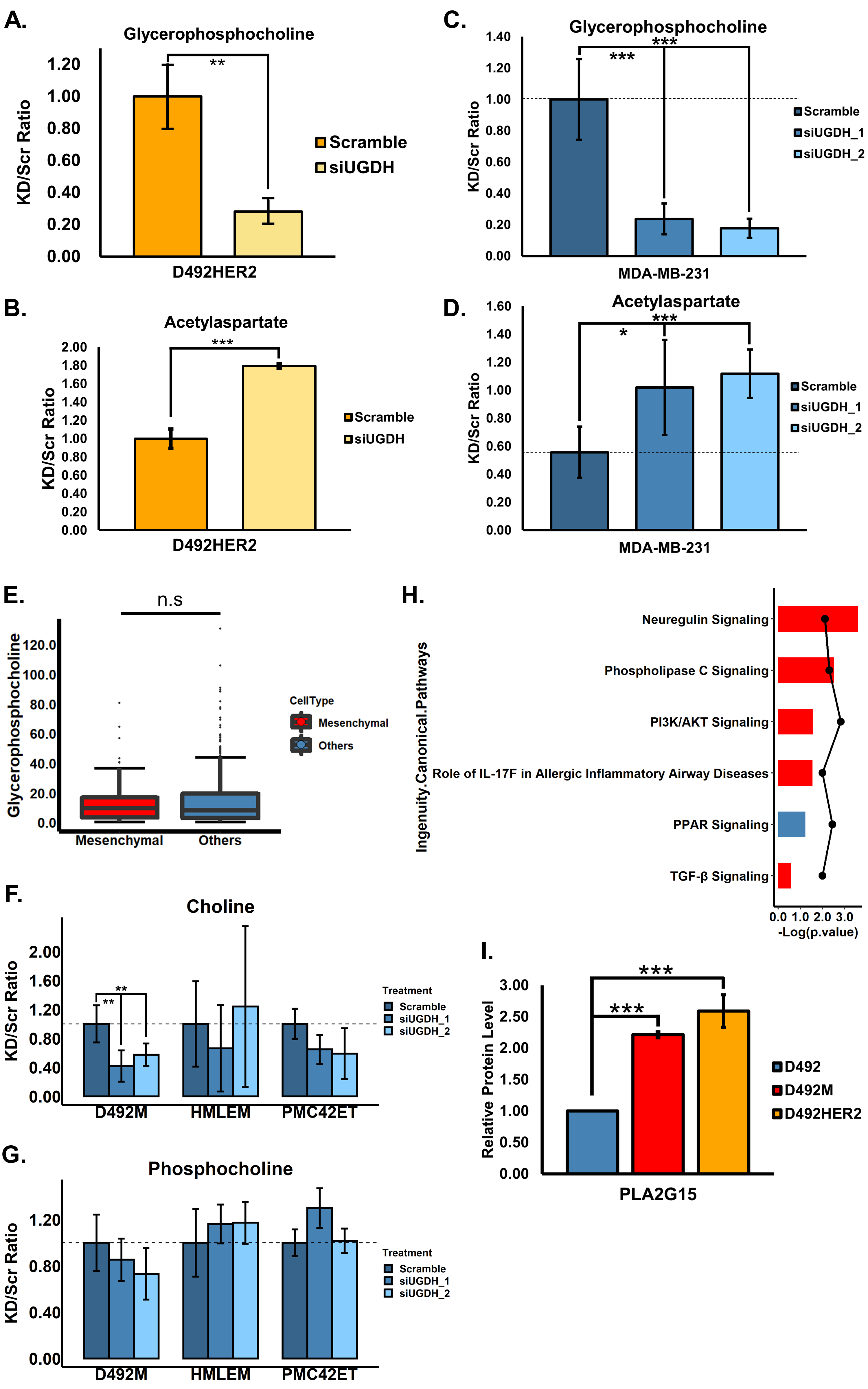

Supplement: Supplementary file 7 — Fig. S7. GPC and NAA were altered with the siUGDH treatment in D492HER2 and MDA‐MB‐231. (A‐D) The glycerophosphocholine (GPC) level was decreased, and the acetylaspartate (NAA) level was increased after the siUGDH treatment in the tumorigenic D492HER2 (n = 3) and malignant MDA‐MB‐231 (n = 6) cell lines. (E) There were no significant differences to the GPC levels between mesenchymal cells and non‐mesenchymal cells based on published datasets in literature [62, 63] (Supplementary Table 5). (F‐G) The expression levels of choline (F) and phosphocholine (G) with UGDH knock‐down in the three EMT cell models. No significant and consistent changes were observed for both metabolites in all the cell lines (n = 5). (H) UGDH has been reported to downregulate PPARγ [27]. To test if there was a negative correlation between UGDH and PPAR signaling, we performed a phosphoproteomic analysis on the D492 EMT cell model (Supplementary Table 7) and noticed that the PPAR signaling was downregulated in the mesenchymal cells where UGDH was highly expressed. The IPA pathways were listed based on the –log10(p value), and the z‐scores for the pathways were represented by the dots/line plot. Red: higher in the mesenchymal D492M; blue: higher in the epithelial D492. (I) The enzyme PLA2G15 potentially involved in the hydrolysis of phosphatidylcholine (PtdCho) into GPC was higher in both D492M and D492HER2. UGDH has been reported to regulate the phosphorylation of ERK (pERK) [32]. cPLA2 is responsible for GPC synthesis from PtdCho in choline metabolism and is under the control of ERK/MAPK [89, 90]. We also observed that PLA2G15 was highly expressed in D492M and D492HER2 compared to D492 (I), suggesting the knock‐down of UGDH may downregulate GPC via pERK‐PLA2G15 (n = 3). Student's T‐test, *: P < 0.05; **: P < 0.01; ***: P < 0.001. n.s: not significant. UGDH, UDP‐glucose 6‐dehydrogenase; PLA2G15, Phospholipase A2 group XV. [file MOL2-16-1816-s012.tif]

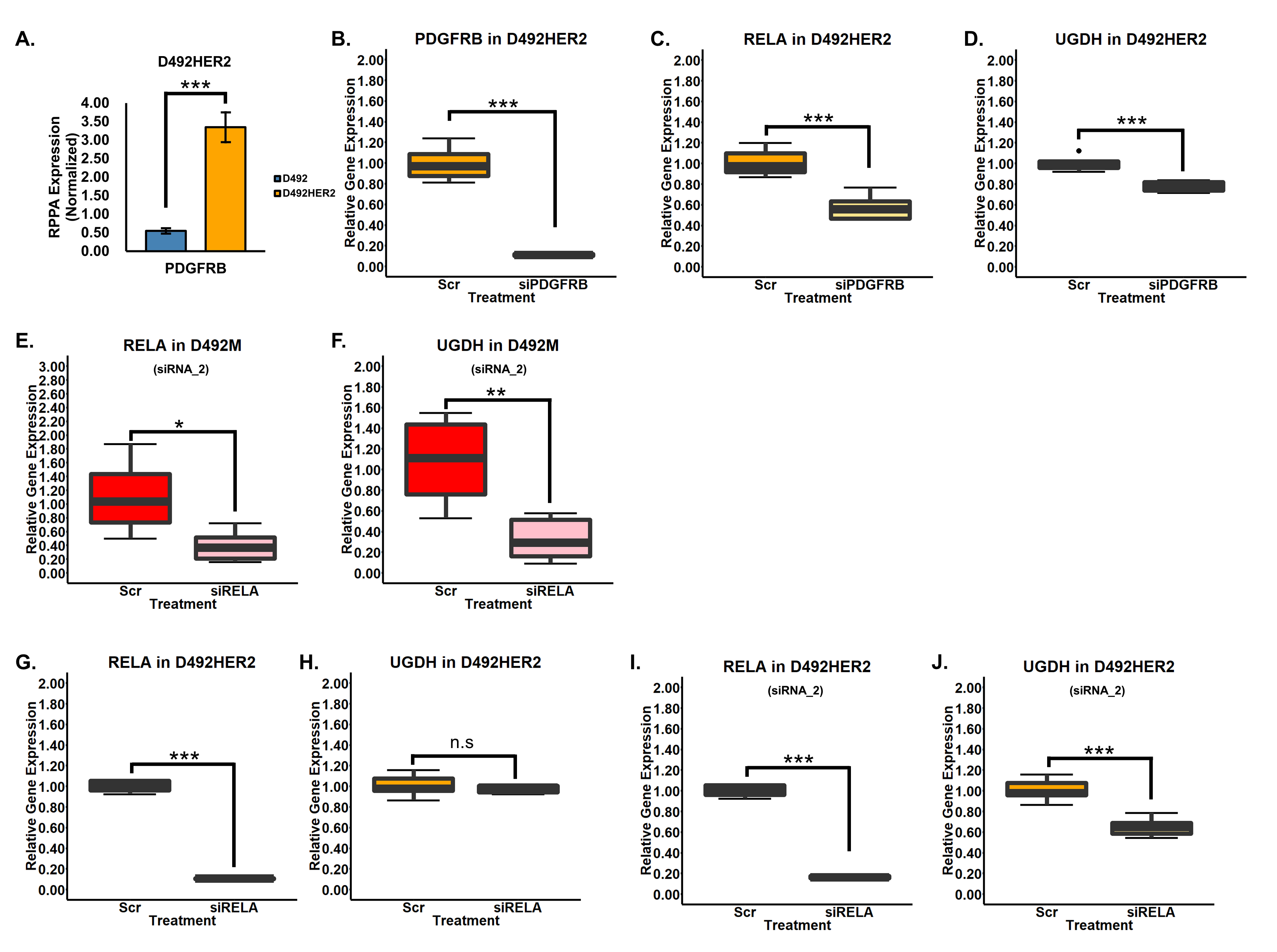

Supplement: Supplementary file 8 — Fig. S8. PDGFRB regulates UGDH via RELA (NFkB‐p65). (A) PDGFRB was highly expressed in the tumorigenic mesenchymal cell line D492HER2 based on the RPPA analysis (n = 3) [65]. (B) The knock‐down efficiency of PDGFRB with siRNA in the D492HER2 cell line was about 90 % (n = 6). (C) RELA (NFkB‐p65) was downregulated after the siRNA knock‐down of PDGFRB in D492HER2 (n = 6). (D) UGDH was downregulated after the siRNA knock‐down of PDGFRB in D492HER2 (n = 6). (E) The knock‐down efficiency of RELA with the second siRNA in D492M was around 70 % (n = 6). (F) UGDH was downregulated after the knock‐down of RELA in D492M with the second siRNA (n = 6). (G) The knock‐down efficiency of RELA with the first siRNA in D492HER2 was around 90 % (n = 6). (H) No significant change in UGDH was observed after the knock‐down of RELA with the first siRNA in D492HER2 (n = 6). (I) The knock‐down efficiency of RELA with the second siRNA in the D492HER2 cell line was about 90 % (n = 6). (J) UGDH was downregulated after the knock‐down of RELA in D492HER2 with the second siRNA (n = 6). Student's T‐test, *: P < 0.05; **: P < 0.01; ***: P < 0.001. UGDH, UDP‐glucose 6‐dehydrogenase; PDGFRB, Platelet‐derived growth factor receptor beta; RELA (NFκB‐p65), Nuclear factor NF‐kappa‐B p65 subunit. [file MOL2-16-1816-s001.tif]
