# Supplementary material for: UDP‐glucose dehydrogenase expression is upregulated following EMT and differentially affects intracellular glycerophosphocholine and acetylaspartate levels in breast mesenchymal cell lines
Source: Mol Oncol. 2022 Feb 3;16(9):1816–40. doi: 10.1002/1878-0261.13172 (PMC9067156; doi:10.1002/1878-0261.13172)
Supplement: Supplementary file 9 — Table S1. The internal standard mix used in the metabolomics analysis. Table S2. A list of primers used in this study. [file MOL2-16-1816-s013.docx]

Supplemental Table 1, 2, and 6

**UDP-glucose dehydrogenase expression is upregulated following EMT and differentially affects intracellular glycerophosphocholine and acetylaspartate levels in breast mesenchymal cell lines.**

Qiong Wang^1^, MSc; Sigurdur Trausti Karvelsson^1^, MSc; Freyr Johannsson^1^, PhD; Arnar Ingi Vilhjalmsson^1^, MSc; Lars Hagen^2,3,4^, PhD; Davi de Miranda Fonseca^2,3,4^, PhD; Animesh Sharma^2,3,4^, PhD; Geir Slupphaug^2,3,4^, PhD; Ottar Rolfsson^1^, PhD

**Affiliations**

^1^Center for Systems Biology, Biomedical Center, Faculty of Medicine, School of Health Sciences, University of Iceland, Sturlugata 8, 101 Reykjavik, Iceland.

^2^Department of Clinical and Molecular Medicine, Norwegian University of Science and Technology, NTNU, N-7491 Trondheim, Norway.

^3^Clinic of Laboratory Medicine, St. Olavs hospital, Trondheim, Norway.

^4^PROMEC Core Facility for Proteomics and Modomics, Norwegian University of Science and Technology, NTNU, and the Central Norway Regional Health Authority Norway.

**Corresponding author information**

Ottar Rolfsson^1^; ottarr@hi.is; tel: +354-5255854; Center for Systems Biology, Biomedical Center, Faculty of Medicine, School of Health Sciences, University of Iceland, Sturlugata 8, 101 Reykjavik, Iceland.

List of Materials

**Supplementary Tables**

**Supplementary Table 1:** The internal standard mix used in the metabolomics analysis.

**Supplementary Table 2:** A List of primers used in this study.

**Supplementary Table 3:** Perseus Output Data *(excel file)*

**Supplementary Table 4:** Raw data of Proteomics *(excel file)*

**Supplementary Table 5:** Publicly available data on the GPC levels of mesenchymal cells *(excel file)*

**Supplementary Table 6:** *In silico* knockdown of UGDH in GEMs.

**Supplementary Table 7:** Data of Phosphoproteomics *(excel file)*

# Supplementary Tables

***Supplementary Table 1.*** The internal standard mix used in the metabolomics analysis.

| **No.** | **Internal Standards** | **Concentrations (µg/mL)** |
| --- | --- | --- |
| 1 | Adenine (^15^N_2_) | 50 |
| 2 | Alanine (d4) | 1000 |
| 3 | AMP (^13^C_10_, ^15^N_5_) | 50 |
| 4 | Arginine (^13^C_6_) | 50 |
| 5 | Carnitine (d9) | 20 |
| 6 | Citric acid (^13^C_6_) | 50 |
| 7 | Cysteine (^13^C_3_, ^15^N) | 50 |
| 8 | Glucose (^13^C_6_) | 2100 |
| 9 | Glutamic Acid (d5) | 30 |
| 10 | Glutamine (^15^N_2_) | 50 |
| 11 | Lysine (d4) | 90 |
| 12 | Malonic acid (d4) | 50 |
| 13 | Octanoic Acid (d15) | 150 |
| 14 | Phenylalanine (d2) | 72 |
| 15 | Phtalic Acid (d4) | 50 |
| 16 | Succinic acid (d4) | 50 |

***Supplementary Table 2.*** A List of primers used in this study.

| **Genes** | **Primers** | **Sequences(5'to3')** |
| --- | --- | --- |
| UGDH | Forward | TTTCTGTGCTGTCCAACCCTGA |
|  | Reverse | CTCTCTGGCCCTCTGGAGTTTC |
| PDGFRB | Forward | GCCGAGCAACTTTGATCAACGA |
|  | Reverse | GCAGTTCTTGGAGGCCAGAAAC |
| RELA | Forward | CCAGACCAACAACAACCCCT |
|  | Reverse | TCACTCGGCAGATCTTGAGC |
| SNAI1 | Forward | ACTATGCCGCGCTCTTTCCT |
|  | Reverse | AGTCCTGTGGGGCTGATGTG |
| ACTB | Forward | CTTCCTGGGTGAGTGGAGACTG |
|  | Reverse | GAGGGAAATGAGGGCAGGACTT |

***Supplementary Table 6.*** *In silico* knockdown of UGDH in the genome-scale metabolic network reconstructions (GEMs) revealed a list of metabolic pathways with changed metabolic fluxes. The GEMs were built on the D492 EMT cell model [25].

| **Group** | **Enriched set size** | **Total set size** | **p-value** | **Adjusted p-value** |
| --- | --- | --- | --- | --- |
| Transport, extracellular | 3 | 859 | 0 | 0 |
| Keratan sulfate degradation | 71 | 74 | 5.25E-101 | 3.94E-100 |
| Keratan sulfate synthesis | 59 | 59 | 1.07E-79 | 5.35E-79 |
| Transport, mitochondrial | 1 | 188 | 2.26E-07 | 8.49E-07 |
| Hyaluronan metabolism | 5 | 5 | 3.38E-06 | 1.01E-05 |
| Transport, lysosomal | 10 | 33 | 1.31E-04 | 3.28E-04 |
| O-glycan synthesis | 4 | 7 | 0.0012 | 0.0025 |
| Glyoxylate and dicarboxylate metabolism | 3 | 6 | 0.0085 | 0.0159 |
| Pentose phosphate pathway | 5 | 27 | 0.0454 | 0.0757 |
| Transport, golgi apparatus | 4 | 25 | 0.0963 | 0.1444 |
| Sphingolipid metabolism | 2 | 17 | 0.2576 | 0.3512 |
| Citric acid cycle | 1 | 19 | 0.334 | 0.3755 |
| Starch and sucrose metabolism | 1 | 8 | 0.3636 | 0.3755 |
| Alanine and aspartate metabolism | 1 | 9 | 0.3755 | 0.3755 |
| Miscellaneous | 1 | 9 | 0.3755 | 0.3755 |
